# Efficient Ventricular Parameter Estimation Using AI-Surrogate Models

**DOI:** 10.3389/fphys.2021.732351

**Published:** 2021-10-14

**Authors:** Gonzalo D. Maso Talou, Thiranja P. Babarenda Gamage, Martyn P. Nash

**Affiliations:** ^1^Auckland Bioengineering Institute, University of Auckland, Auckland, New Zealand; ^2^Department of Engineering Science, University of Auckland, Auckland, New Zealand

**Keywords:** optimisation, cardiac mechanics, surrogate model, MLP, parameter estimation

## Abstract

The onset and progression of pathological heart conditions, such as cardiomyopathy or heart failure, affect its mechanical behaviour due to the remodelling of the myocardial tissues to preserve its functional response. Identification of the constitutive properties of heart tissues could provide useful biomarkers to diagnose and assess the progression of disease. We have previously demonstrated the utility of efficient AI-surrogate models to simulate passive cardiac mechanics. Here, we propose the use of this surrogate model for the identification of myocardial mechanical properties and intra-ventricular pressure by solving an inverse problem with two novel AI-based approaches. Our analysis concluded that: (i) both approaches were robust toward Gaussian noise when the ventricle data for multiple loading conditions were combined; and (ii) estimates of one and two parameters could be obtained in less than 9 and 18 s, respectively. The proposed technique yields a viable option for the translation of cardiac mechanics simulations and biophysical parameter identification methods into the clinic to improve the diagnosis and treatment of heart pathologies. In addition, the proposed estimation techniques are general and can be straightforwardly translated to other applications involving different anatomical structures.

## 1. Introduction

Cardiovascular disease is the largest cause of death worldwide. Effective diagnosis and treatment are hampered by a lack of knowledge of the pathophysiological mechanisms underlying the development of the disease. Biomechanical factors, such as stiffness and stress, are known to have important influences on heart function, but are difficult to quantify. Patient-specific computer models of heart biomechanics allow intrinsic constitutive muscle properties, including stiffness, contraction, relaxation, stress and work, to be assessed using medical data from cardiac catheterisation and imaging (Wang et al., 2018). Such cardiac tissue indices provide a new dimension of diagnosis that can help to elucidate the mechanisms of heart disease, thus enabling more specific targeting of treatment and ultimately better outcomes for patients.

Simulating biomechanics via computational models of the heart is challenging due to the stress-strain non-linearities intrinsic of cardiac tissue undergoing large deformation. The finite element method (FEM) is the most commonly used approach to solve the finite elasticity governing equations to enable accurate predictions of these large deformations (Nash and Hunter, 2000). This requires specifying constitutive relations to describe the stress-strain behaviour of the myocardium. Calibration of patient-specific parameters of these constitutive relations typically involves minimising an objective function that quantifies kinematic discrepancies between the FE model's predictions and measurements from medical images. These kinematic measurements typically involve quantifying shape change or the displacement of the myocardium over the cardiac cycle. For example, segmentations of the endocardial and epicardial contours of the heart from Cine MRI (Chen et al., 2020) have been used as shape-based kinematic measurements for FE model calibration (Wang et al., 2018). Kinematic measurements that involve quantify the displacement of the heart wall have also been used for FE model calibration (Hadjicharalambous et al., 2017; Zhang et al., 2021). These approaches track the displacements of the tissue using techniques such as optical flow (Queiros et al., 2017) or cardiac magnetic resonance tagging (Aletras et al., 1999; Zhong et al., 2010; Ibrahim, 2011; Shi et al., 2012; Amzulescu et al., 2019).

A number of non-linear optimisation methods have been used to calibrate constitutive parameters of cardiac mechanics models. This includes the application of gradient-based methods (Gao et al., 2015; Wang et al., 2018) and gradient-free methods (Rumindo et al., 2020; Zhang et al., 2021). Each of these methods requires multiple evaluations of the objective function for each update of the parameters, and each evaluation involves a costly FE simulation of the mechanics model. The evaluation of the gradient is often obtained from finite difference approximations, which can involve a significant computational cost when increasing number of parameters need to be identified. Calibration procedures using gradient-based or gradient free methods are therefore computationally expensive, often taking many hours or days to complete [e.g., (Gao et al., 2015) reported calibration times of 63 h, and (Zhang et al., 2021) reported calibration times of 15 h]. This presents a significant barrier to clinical translation of cardiac mechanics models.

Surrogate models have been developed to improve simulation efficiency, replacing computationally expensive FE simulations with inexpensive surrogate simulations (Dabiri et al., 2019; Maso Talou et al., 2020; Cai et al., 2021). These surrogate models have also been used to accelerate calibration procedures (Di Achille et al., 2018; Davies et al., 2019; Noe et al., 2019; Longobardi et al., 2020; Cai et al., 2021). For example, Cai et al. (2021) presented an approach that used surrogate models with a trust-region-reflective gradient-based optimiser for estimating personalised constitutive parameter of the left ventricle. While these advancements are promising, existing calibration approaches do not exploit the full potential of neural networks-based surrogate models, which can directly provide the analytic gradients of objective functions of interest during the calibration procedure via automatic differentiation (Raissi et al., 2019). By using these analytic gradients, we only need to evaluate the model once for each iteration of gradient-based optimisation procedures. This can lead to an efficient personalisation of these cardiac models, and enable their future application to real-time continuous monitoring of cardiac function.

In this work, we propose a novel approach that uses AI-surrogate model and automatic differentiation to efficiently identify constitutive parameters or loading conditions of a biomechanical model given kinematic measurements from medical images. This builds upon our recent work in developing deep learning approaches that substantially reduce the computational cost of simulating cardiac biomechanics, by training an AI-surrogate model that accurately reproduces mechanics predictions with a fraction of the computational cost of numerical methods that solve the governing partial differential equations (Maso Talou et al., 2020). We demonstrate this approach for estimating passive stiffness or pressure of the left ventricle (LV). This approach involves performing an optimisation of the AI-surrogate inputs to best match its kinematic response against a set of given observations from medical images. We present two different strategies: (i) a full-field tracking approach, which requires the displacement field between two medical images; and (ii) a contour matching approach, which requires only the geometry of the ventricular surfaces in between two medical images. The latter approach relaxes the requirement for determining the displacement field of the tissue, and only requires contours describing the surface of the ventricle from medical images to determine the best matching kinematic response of the AI-surrogate.

The manuscript is structured as follows. In section 2, we introduce the FE mechanical model for the left ventricle of the heart, and its AI-surrogate. Then, we present the proposed parameter identification strategies followed by the setup of the optimisation scheme. In section 3, we study the performance of both strategies for the identification of constitutive parameters and haemodynamic loading conditions. Finally, we discuss the contributions and limitations of this work in section 4 and outline our final remarks in section 5.

## 2. Methods

### 2.1. Mechanical Model

Kinematics of the LV are simulated using a patient-specific FE model (Wang et al., 2018). This involves solving the finite elasticity equilibrium equations during the diastolic phase of the cardiac cycle under an endocardial pressure boundary condition to simulate passive filling. Patient-specific geometrical models of the LV are constructed at the diastasis frame of the cardiac cycle for a range of individuals, which are assumed to be in a load-free configuration. Cubic Lagrange basis functions are used for constructing the FE mesh of the geometry. A typical mammalian description of the myocyte orientation through the LV wall (Nielsen et al., 1991) is incorporated into the geometry through a material fibre field. The LV myocardium is modelled as an ideally-incompressible transversely isotropic material by means of a Fung-type exponential constitutive model (Guccione et al., 1991) with the following strain energy density function


(1) $$\begin{array}{l} \begin{array}{l} \text{Ψ}=\frac{c_{1}}{2}\left(e^{Q}-1\right)\\ Q=c_{2}E_{ff}^{2}+c_{3}\left(E_{cc}^{2}+E_{rr}^{2}+E_{cr}^{2}\right)+2c_{4}\left(E_{fc}^{2}+E_{fr}^{2}\right) \end{array} \end{array}$$

where the *c*_1_ parameter scales the overall stiffness of the myocardium, and *c*_2_, *c*_3_, and *c*_4_ control the material non-linearity and anisotropy in the fibre (f), cross-fibre (c), and radial (r) directions, respectively. Incompressibility of the myocardium was enforced through a mixed formulation that uses linear Lagrange basis functions for describing the hydrostatic pressure (Nash and Hunter, 2000). Homogeneous Dirichlet boundary conditions were applied on nodes of the FE mesh at the epicardial perimeter of the basal surface of the model. All FE simulations were performed using the OpenCMISS-Iron open-source computational modelling software package (Bradley et al., 2011).

### 2.2. Surrogate Network Model

From the FE mechanical model defined in section 2.1, we derive a surrogate model as described in Maso Talou et al. (2020). The AI-surrogate model predicts the displacement of a material point **x** = (*x*_*d*_, *y*_*d*_, *z*_*d*_) for a given intra-ventricular pressure *p*, domain description **g** = (*g*_1_, *g*_2_) (PCA weights as explained in Maso Talou et al., 2020) and the constitutive parameters (*c*_1_, *c*_2_). Particularly, we fixed parameters *c*_3_ = 3.67 and *c*_4_ = 25.77 as their identifiability from macro-scale observations is low.

To train the AI-surrogate, we minimised the squared displacement error with the FE predictions given by training the following loss function


(2) $$\begin{array}{l} l\left(B\right)=\underset{{\text{u}}_{d}\in B_{d}}{\sum }∥{\text{u}}_{d}-{\tilde{\text{u}}}_{d}{∥}_{L_{2}}^{2}+\alpha \underset{{\text{u}}_{b}\in B_{b}}{\sum }∥{\text{u}}_{b}-{\tilde{\text{u}}}_{b}{∥}_{L_{2}}^{2} \end{array}$$

where $B_{b}$ and $B_{d}$ define sets of points (training batches) on the basal/endocardial boundary (where Dirichlet or Neumann boundary conditions were applied) and inside the domain, respectively, **u**_*i*_ and ${\tilde{\text{u}}}_{i}$ are the displacements predicted with the neural network and the FE model, respectively, α = 4.5 is the penalty factor to impose the boundary conditions, and $B=B_{b}+B_{d}$ is a given training batch. For further details about the training of the AI-surrogate, refer to (Maso Talou et al., 2020).

### 2.3. Parameter Identification Strategies

In this work, we propose two strategies to identify inputs of the surrogate model from a given set of observations of ventricular kinematics from medical images. As presented in our previous work (Maso Talou et al., 2020), we can encode boundary conditions, applied tractions, domain geometry and constitutive parameters as inputs of these networks, thus the proposed techniques can be used to characterise any of these inputs.

The estimation of parameters is studied using two approaches, which differ based on the image data available to quantify the kinematics of the heart. One approach considers a displacement field description of the cardiac wall throughout the cardiac cycle, which can be derived from medical images using motion tracking methods (Wang and Amini, 2011; Shi et al., 2012), or post-processing functions for MR sequences that provide effective tracking of material points (e.g., CMR tagging). For the second approach, the input data describe the kinematics of the cardiac wall surfaces during the heart cycle. For each approach, two different time-points, corresponding to the initial and final positions of the cardiac walls, were combined with the measured intra-ventricular pressures that obtained from cardiac catheterisation for the analyses. Quantification of the cardiac wall surfaces can be obtained through segmentation of clinical images, such as 3D echocardiography or cardiovascular magnetic resonance imaging (CMR) (Chen et al., 2020).

#### 2.3.1. Full-Field Tracking Approach

The full-field tracking approach involves finding the parameters Θ (inputs of the surrogate model) that minimise the error between the surrogate model's predictions of tissue motion and displacement data derived from medical images, i.e.,


(3) $$\begin{array}{l} \Theta =\underset{\hat{\Theta }}{arg min}L\left(\hat{\Theta },\hat{\text{u}}\right)\\ \;=\underset{\hat{\Theta }}{arg min}∥\text{u}\left(\hat{\Theta }\right)-\hat{\text{u}}{∥}_{L_{2}} \end{array}$$

where $u\left(\hat{\Theta }\right)$ is the displacement field predicted by the surrogate model for parameters $\hat{\Theta }$, and $\hat{\text{u}}$ is the displacement field from medical images.

#### 2.3.2. Contour Matching Approach

Let us define a contour **s**_*t*_ at the time-point *t* composed of *P* points as ${\text{s}}_{t}=\left({\text{x}}_{t}^{0},\ldots ,{\text{x}}_{t}^{P}\right)$. The endocardial and epicardial contours are extracted from medical images at the reference (diastasis) and pressure loaded (end-diastolic) time-points. We denote these contours as *s*^endo^ and *s*^epi^ for endocardial and epicardial contours, respectively, and in the following we use subscripts *i* or *f* to indicate the absence or presence, respectively, of pressure load.

The contour matching approach involves finding the parameters Θ (inputs of the surrogate model) that minimise the error between the initial contours ($s_{i}^{\cdot }$) displaced by surrogate model predictions, and the end-diastolic contours identified from the medical images, namely


(4) $$\begin{array}{l} \Theta =\underset{\hat{\Theta }}{arg min}L\left(\hat{\Theta },{\text{s}}_{i}^{\text{endo}},{\text{s}}_{f}^{\text{endo}},{\text{s}}_{i}^{\text{epi}},{\text{s}}_{f}^{\text{epi}}\right)\\ =\underset{\hat{\Theta }}{arg min}\left(d\left({\text{s}}_{i}^{\text{endo}}+\text{u}\left(\hat{\Theta }\right),{\text{s}}_{f}^{\text{endo}}\right)+d\left({\text{s}}_{i}^{\text{epi}}+\text{u}\left(\hat{\Theta }\right),{\text{s}}_{f}^{\text{epi}}\right)\right)\; \end{array}$$

where the surface-field addition yields a predicted surface ${\text{s}}_{t}+\text{u}=\left({\text{x}}_{t}^{0}+\text{u}{|}_{{\text{x}}_{t}^{0}},\ldots ,{\text{x}}_{t}^{p}+\text{u}{|}_{{\text{x}}_{t}^{p}}\right)$ with **u**|_**x**_ being the displacement of the cardiac wall at the spatial position **x**, and


(5) $$\begin{array}{l} d\left({\text{s}}_{1},{\text{s}}_{2}\right)=\frac{1}{\left|{\text{s}}_{1}\right|}\underset{\text{x}\in {\text{s}}_{1}}{\sum }\underset{\text{y}\in {\text{s}}_{2}}{\min}∥\text{x}-\text{y}∥ \end{array}$$

where ∥·∥ is the Euclidean norm and |**s**| is the cardinality of **s**.

### 2.4. Optimisation Scheme

A surrogate model of the ventricle is obtained as described in Maso Talou et al. (2020) by minimising a displacement error metric (see Equation 2) between the model predictions and a finite element model. During the training of the AI-surrogate, both the training and testing errors decreased monotonically across epochs, reaching a plateau at 4.43 × 10^−4^ mm^2^ and 1.30 × 10^−3^ mm^2^, respectively. The resulting surrogate model presents an absolute displacement error of 0.0499 ± 0.0374 mm in ranges of intraventricular pressure (*p* ∈ [0.15, 1.5] kPa) and myocardial elasticity (*c*_1_ ∈ [2, 5] kPa and *c*_2_ ∈ [4, 40]) within the physiological ranges.

As the parameters to be estimated are inputs of surrogate model's network, we freeze the weights of the network layers and optimise the inputs by solving Equations (3) or (4). By using Tensorflow v2.1, we implemented the network and the objective function in a Tensorflow graph. Via this execution graph and automatic differentiation, we obtain the analytic derivatives of Equations (3) or (4) with respect to the parameters to be estimated. Finally, we employ the derivatives in an ADAM method with exponential decay of the learning rate given by


(6) $$\begin{array}{l} \tau_{i}=\tau_{0}0.985^{i/10} \end{array}$$

where τ_*i*_ is the learning at the *ith* epoch, and τ_0_ is the initial learning rate. We empirically choose τ_0_ = 1 for both full-field tracking and contour matching approaches. By using learning decay, the optimisation proceeds more rapidly in the neighbourhood of the target value, and the overshooting oscillations are damped, as the learning rate diminishes, leading to convergence. The optimisation process stops when estimates of all of the parameters show a relative change lower than 10^−5^ in the last epoch, i.e.,


(7) $$\begin{array}{l} {\left\|\frac{\Theta_{n+1}-\Theta_{n}}{\Theta_{ref}}\right\|}_{\infty }<10^{-5} \end{array}$$

where Θ_*i*_ is the estimate of the parameters at the *ith* epoch, Θ_*ref*_ is a vector with reference values for each parameter, and vector division is the element-wise division of the vector components.

## 3. Results

**Figure 1:**
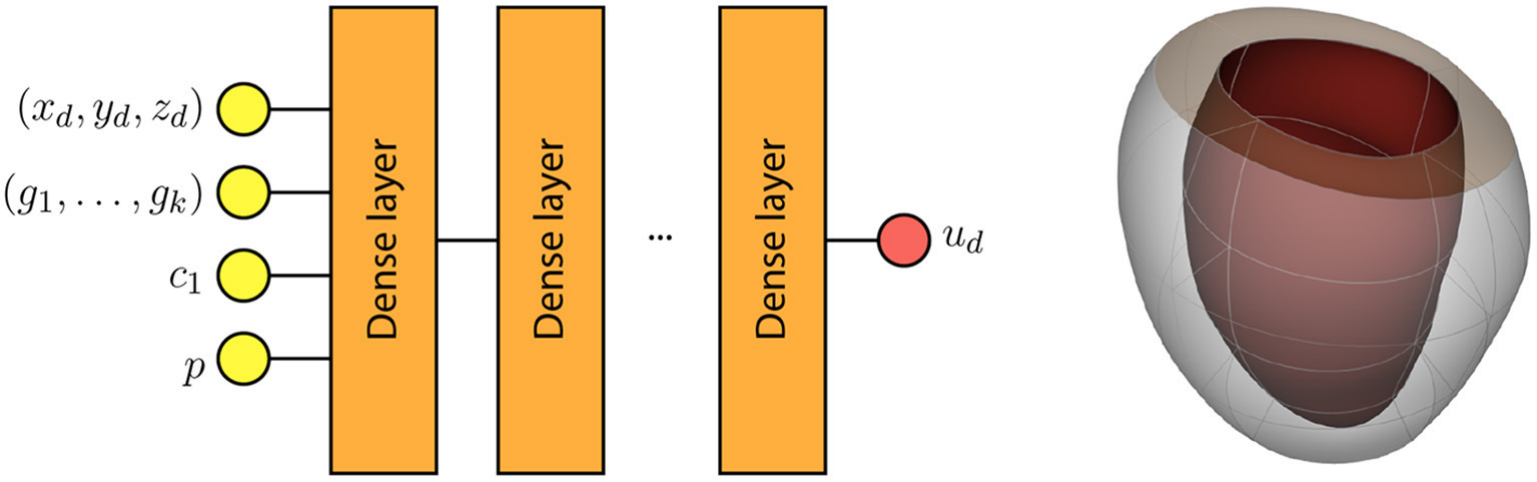
**(left)** AI-surrogate depicting its inputs and outputs (Maso Talou et al., 2020); **(right)** Example of the geometry of the ventricle at diastasis for **g** = **0**.

In this section, we analyse the ability of both formulations (see Equations 3 and 4) to identify myocardial constitutive parameters, and the intra-ventricular pressure. In our assessment, we disregard the representation error of the domain associated with the PCA and learning space presented in Maso Talou et al. (2020), as it can be reduced by including additional PCA modes as inputs of our surrogate model. Without loss of generality, we fixed the geometry to **g** = **0** (i.e., mean ventricular shape across our population, see Figure 1). The analyses reported here will be analogous for different geometrical variations of the left ventricle as well as for different Dirichlet boundary conditions. All observations $\hat{\text{u}}$, ${\text{s}}_{f}^{\text{endo}}$ and ${\text{s}}_{f}^{\text{epi}}$ in this work have been generated using the same FE model (see section 2.1) to train the surrogate model, thus there is no model error in this study.

Additionally, we studied the effect of tracking and segmentation errors (observation errors) on the estimates of interest. The observation error is modelled as


(8) $$\begin{array}{l} {\text{u}}_{\epsilon }=\frac{\text{r}}{∥\text{r}∥}\eta \end{array}$$

where **r** is a randomly oriented vector with components $r_{i}\in U\left(0,1\right)$ and η∈N(0,σ) is the magnitude of **u**_ϵ_. Note that the magnitude of our error is normally distributed with zero-mean, and we choose σ = 1 mm to represent errors of similar magnitude to the image resolution of MRI images for myocardial structures. This error is added to $\hat{\text{u}}$, ${\text{s}}_{f}^{\text{endo}}$, and ${\text{s}}_{f}^{\text{epi}}$ for the cases with noise.

Regarding spatial discretisation of the observations (i.e., the number of samples for $\hat{\text{u}}$, ${\text{s}}_{f}^{\text{endo}}$, and ${\text{s}}_{f}^{\text{epi}}$), we sampled 1, 109 points across the cardiac wall and 7, 579 points over each of the ventricular surfaces to describe $\hat{\text{u}}$ and ${\text{s}}_{f}^{\cdot }$, respectively. In turn, for the spatial discretisation of the AI-surrogate inputs, we sampled 1, 109 points across the cardiac wall for the full-field tracking approach and 1, 072 points to describe each of the surfaces ${\text{s}}_{i}^{\cdot }$. By oversampling ${\text{s}}_{f}^{\cdot }$ with respect to ${\text{s}}_{i}^{\cdot }$, we can compute the distance between these surfaces by substituting ${\text{s}}_{1}={\text{s}}_{f}^{\cdot }$ into Equation (5). This approximation yields a reasonable trade-off between computation time and discretisation error (note that the discretisation error decreases as |**s**_1_| increases).

### 3.1. Identification of Constitutive Parameters

We first studied the precision of simultaneously estimating the constitutive parameters *c*_1_ and *c*_2_ for a known intra-ventricular pressure *p*. The estimates were generated for both approaches under three experimental scenarios: (i) a two time-point experiment without observation error, i.e., only two images are available during the cardiac cycle; (ii) a two time-point experiment with observation error; and (iii) a multiple time-point (*N* = 10) experiment with observation error. The first two experiments illustrate the degradation of *c*_1_ and *c*_2_ estimates for both approaches as measurements become less reliable due to observation errors. This indicates the robustness of the approaches with respect to observation error. A comparison of the last two experiments shows the benefits of including additional observations (time-points), which diminish the impact of the stochastic component of the observation error.

#### 3.1.1. Experiment 1

**Figure 2:**
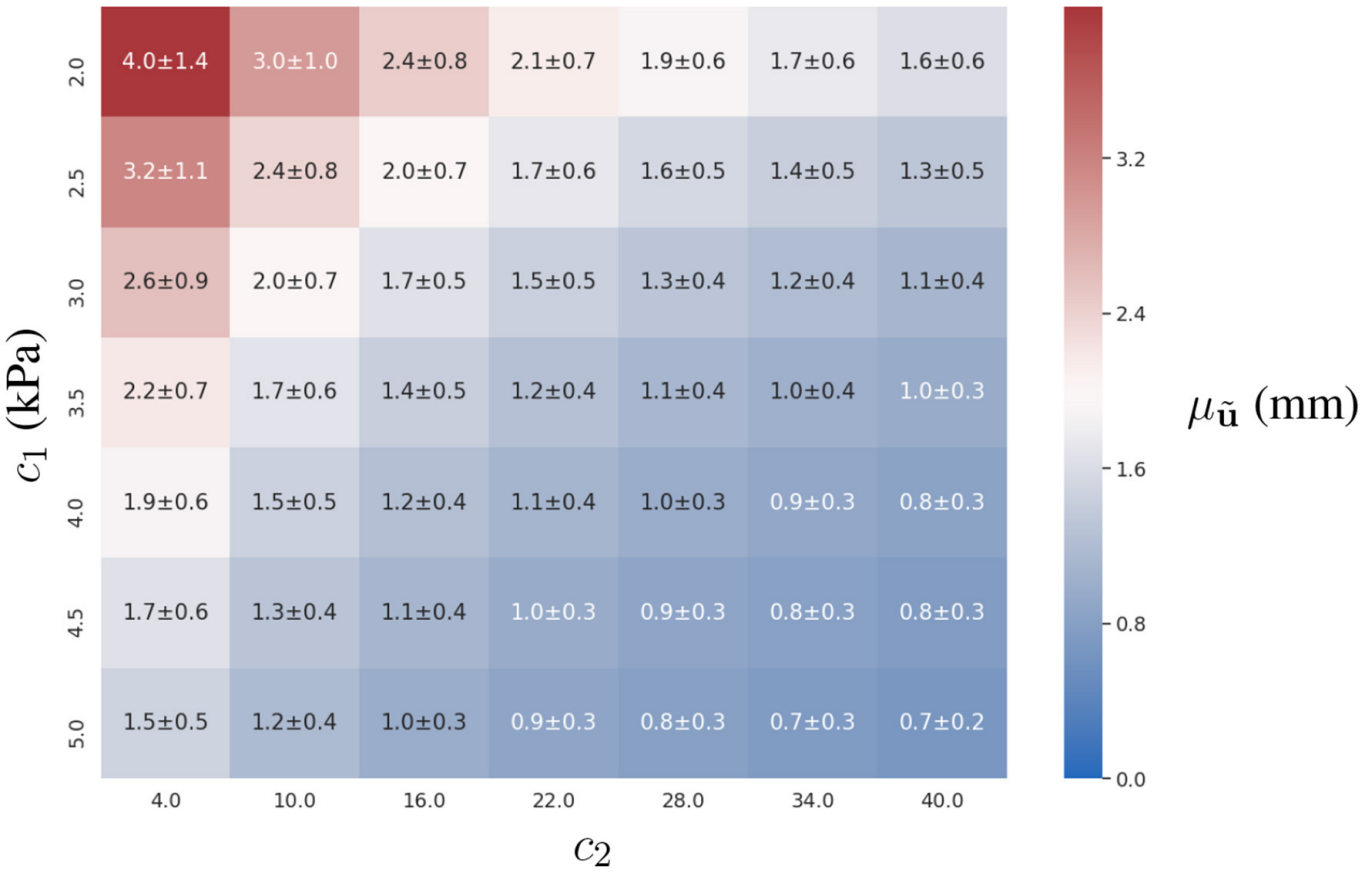
Mean displacement of the ventricular wall (denoted $\mu_{\hat{\text{u}}}$) for different combinations of the constitutive parameters *c*_1_ and *c*_2_, and an intra-ventricular pressure load of *p* = 0.9 kPa. For details of mean displacements for other loads, refer to the Supplementary Material.

**Figure 3:**
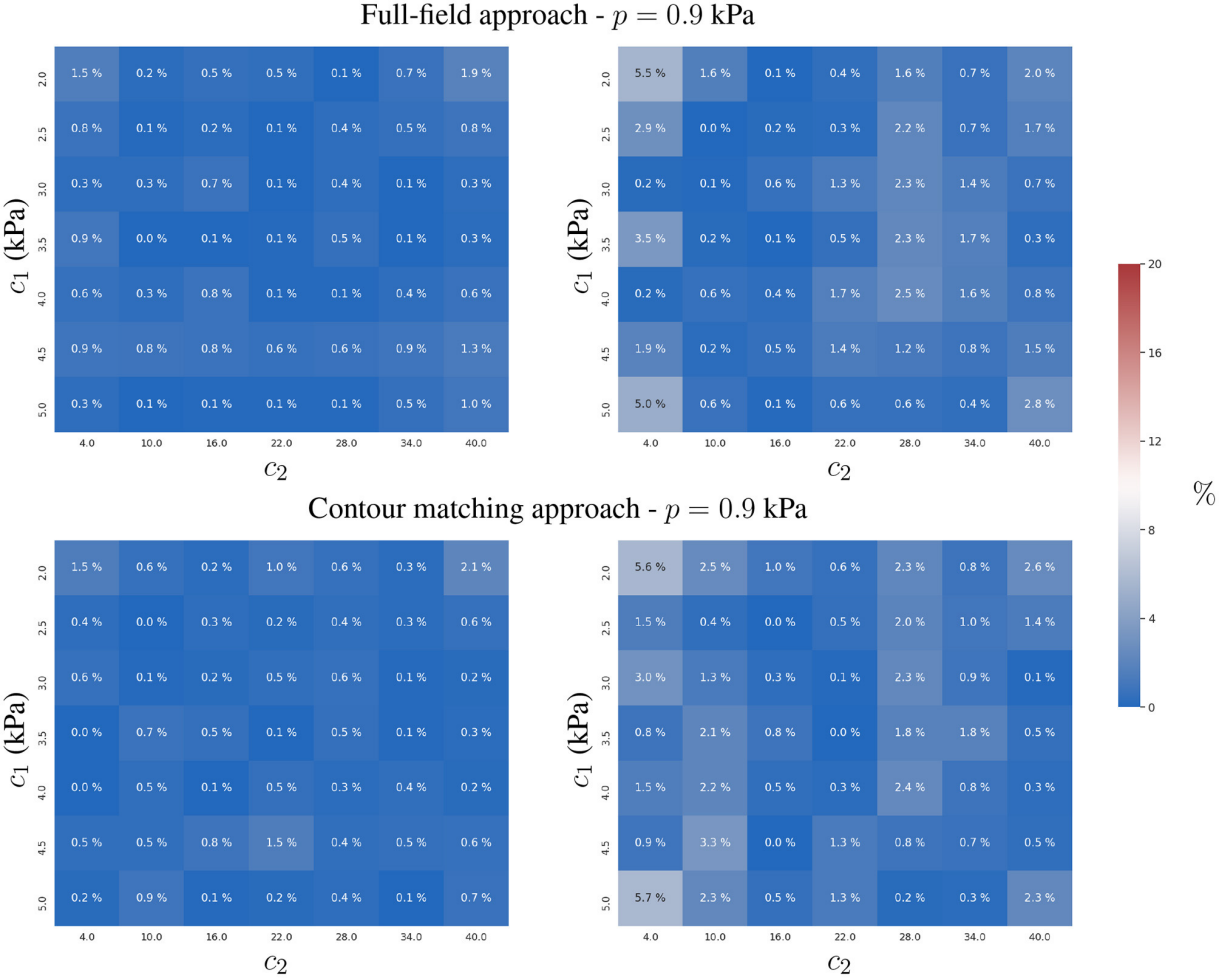
Relative error of the two-parameter estimation. Estimates of the constitutive parameters *c*_1_ **(left)** and *c*_2_ **(right)** are presented using the full-field tracking and contour matching approaches with a load of *p* = 0.9 kPa in the absence of noise in the observations. For details of estimations for other loads, refer to the Supplementary Material.

Both approaches showed a similar accuracy in the absence of observation errors. Since variations in the predicted displacements are more sensitive with respect to *c*_1_ than *c*_2_, we observed better estimates of *c*_1_ (see **Figure 3**). The distribution of the error in the parametric space (*c*_1_, *c*_2_) does not show an association between the displacement magnitudes and the estimation error (compare Figures 2, 3).

#### 3.1.2. Experiment 2

**Figure 4:**
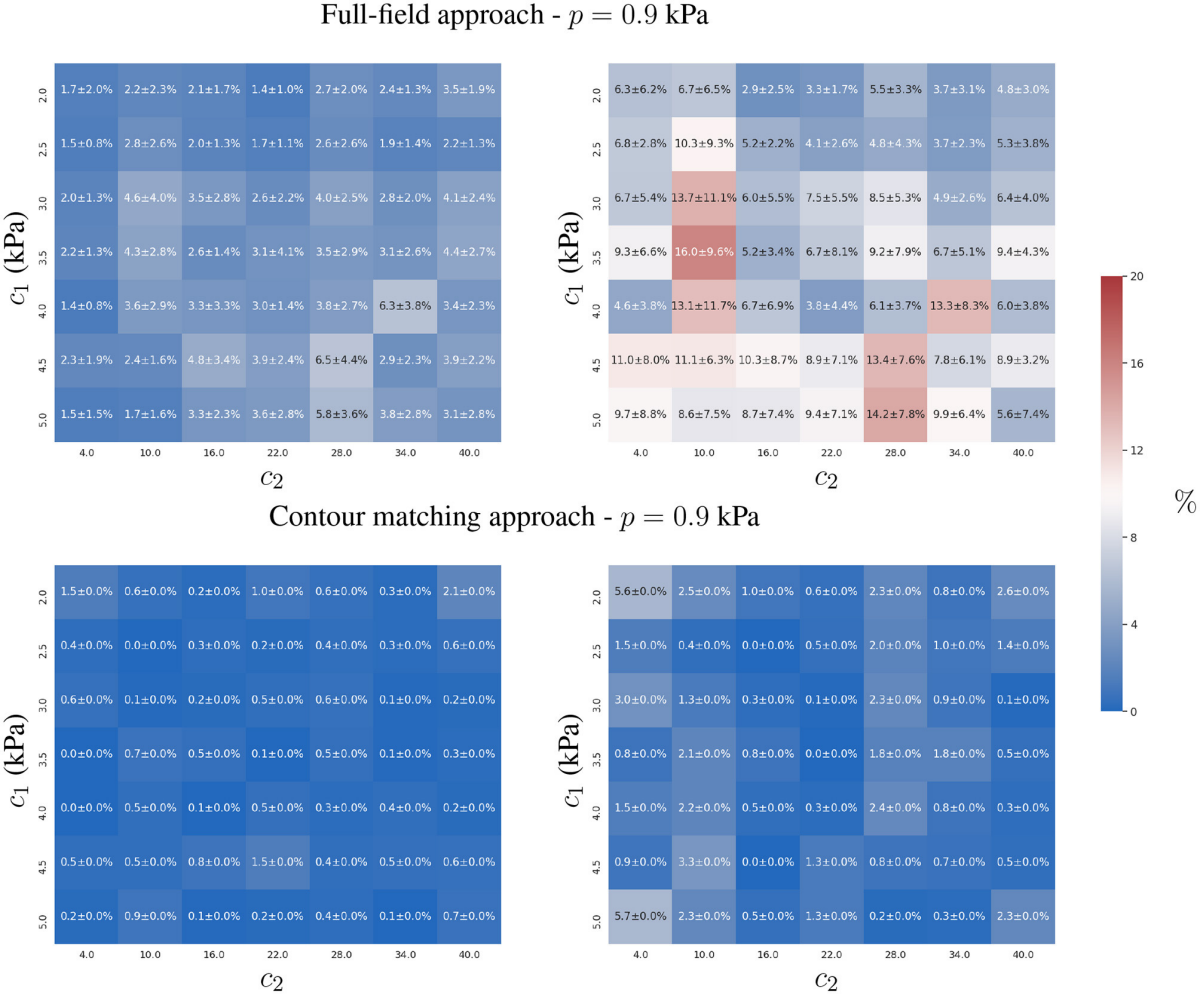
Mean and standard deviation of the relative error for the two-parameter estimation of *c*_1_ **(left)** and *c*_2_ **(right)** constitutive parameters using the full-field tracking and contour matching approaches with a load of *p* = 0.9 kPa and the presence of Gaussian noise (σ = 1mm ≈ 2 pixels) in the observations. For details of estimations for other loads, refer to the Supplementary Material.

To analyse the effect of observation errors, we repeated the previous study 10 times while adding different independent instances of **u**_ϵ_ (see Equation 8) to the observations $\hat{\text{u}}$, ${\text{s}}_{f}^{\text{endo}}$ and ${\text{s}}_{f}^{\text{epi}}$. To quantify the effect of the noise on the estimates across these different samples, we computed the mean and standard deviation of the relative error when estimating the parameters *c*_1_ and *c*_2_ (see Figure 4).

The contour matching approach provided more accurate estimates than the full-field tracking approach in the presence of observation errors (see Figure 4). This is shown by the lower overall error of the contour matching approach, which is insensitive to displacements within the muscular heart wall, and within the surfaces of the ventricles. As only a small tangential component of displacement is expected in the endocardial and epicardial surfaces from the loading process in our FE model, the insensitivity toward such component is beneficial for the estimation process. Note that this may not be true in practical scenarios, where the quantification of such components is yet to be thoroughly explored.

The full-field tracking approach showed larger estimation errors for *c*_1_ in stiffer materials. In such cases, the displacement-to-**u**_ϵ_ ratio (analogous to the signal-to-noise ratio in signal processing) was smaller, hindering the estimation due to the increasing effects of noise in the observations. *c*_2_ presented lower identifiability due to the plateau in the objective function, L(Θ), associated with the observation error (see Equation 3 and **Figure 9**). The sensitivity of *c*_2_ with respect to the displacements was higher than that of *c*_1_ due to the form of the constitutive equation (Equation 1). The identifiability of *c*_2_ is susceptible to surrogate approximation errors, i.e., discrepancies between the AI-surrogate and the FE model. An example of this, is the increase in error of *c*_2_ as shown in Figure 4. The AI-surrogate underestimates ∂**u**/∂*c*_2_ in its predictions contributing to the plateau in L(Θ) described previously. Note that, as the pressure is increased the AI-surrogate error has a diminishing effect on the predicted displacement (see Supplementary Figures 7, 8).

#### 3.1.3. Experiment 3

We analysed the effect of adding multiple time-points for the estimation of the constitutive parameters when observation errors were present. We assumed that the observation errors, **u**_ϵ_, for all time-points, were independent and identically distributed (uncorrelated).

**Figure 5:**
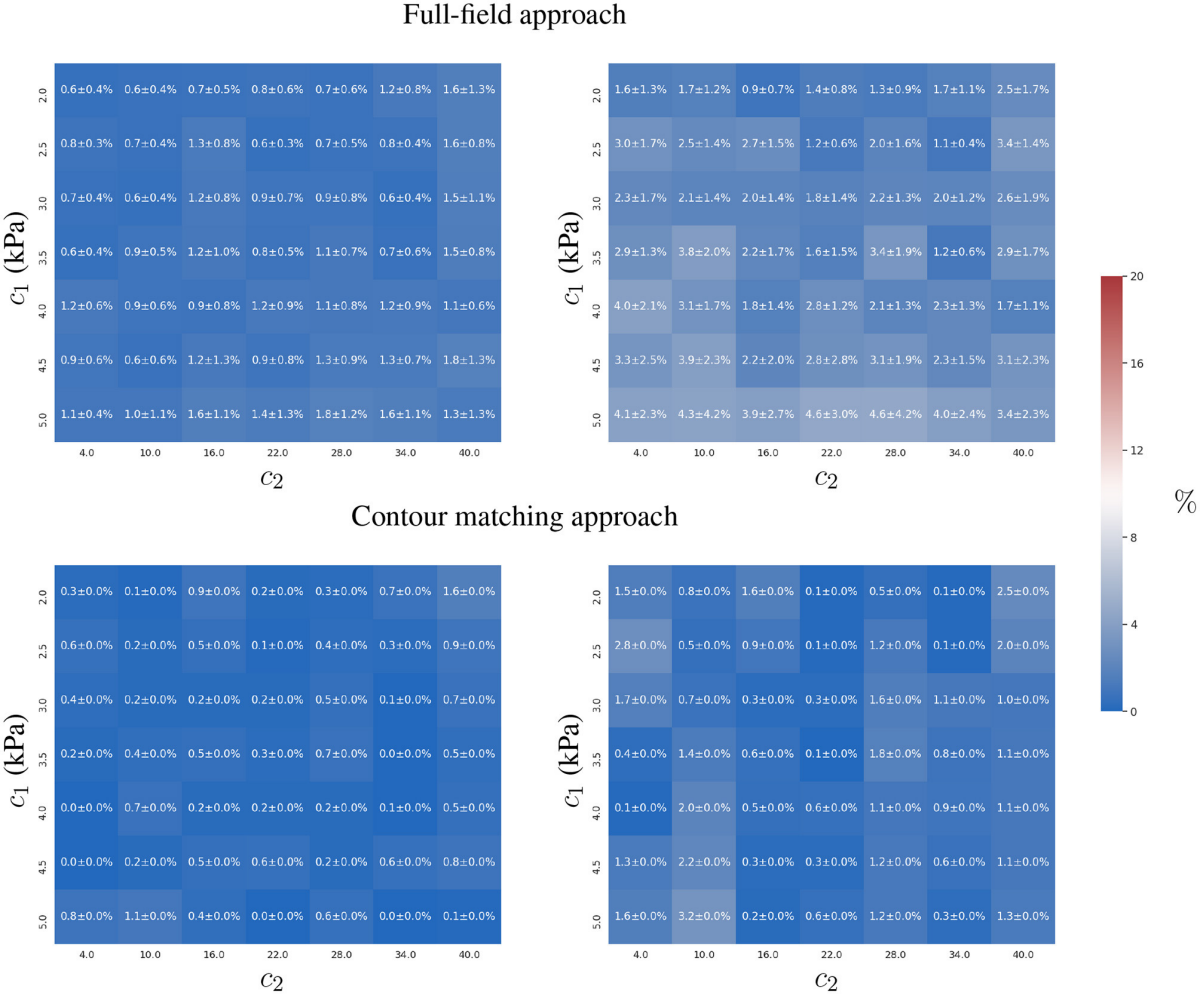
Mean and standard deviation of the relative error for the two-parameter estimation of *c*_1_ **(left)** and *c*_2_ **(right)** constitutive parameters using full-field tracking and contour matching approaches, a pressure trace with 10 time-points, and independent Gaussian noise (σ = 1mm ≈ 2 pixels) in the observations.

We observed that both approaches showed an improvement in reducing the mean relative error in the estimation of *c*_1_ and *c*_2_ (see Figure 5), closer to values from error-free estimates reported in Figure 3. As the observation error is normal, zero-mean and independent between the time-points, both formulations efficiently cope with the uncertainty, because the Euclidean norm involved in both formulations (Equations 3 and 5) optimises toward the mean of the error distribution.

The contour matching approach is slightly more accurate, mainly in the estimation of *c*_2_ (maximum error of 3%, in comparison to the full-field tracking approach, with a maximum error of 5 ± 4 %).

### 3.2. Identification of Intra-Ventricular Pressure

The previous section studied the accuracy of estimating the constitutive parameters, *c*_1_ and *c*_2_, given a known intra-ventricular pressure, *p*. In this section, we analogously analyse the accuracy in the estimation of *p*, assuming that the constitutive parameters are known. Two experiments were conducted: (i) a two time-point experiment without observation error where only two images are available; and (ii) a two time-point experiment with observation error. Analogous to the previous section, we studied the degradation in the ability to recover the intra-ventricular pressure for both approaches as the observations become less reliable.

#### 3.2.1. Experiment 1

**Figure 6:**
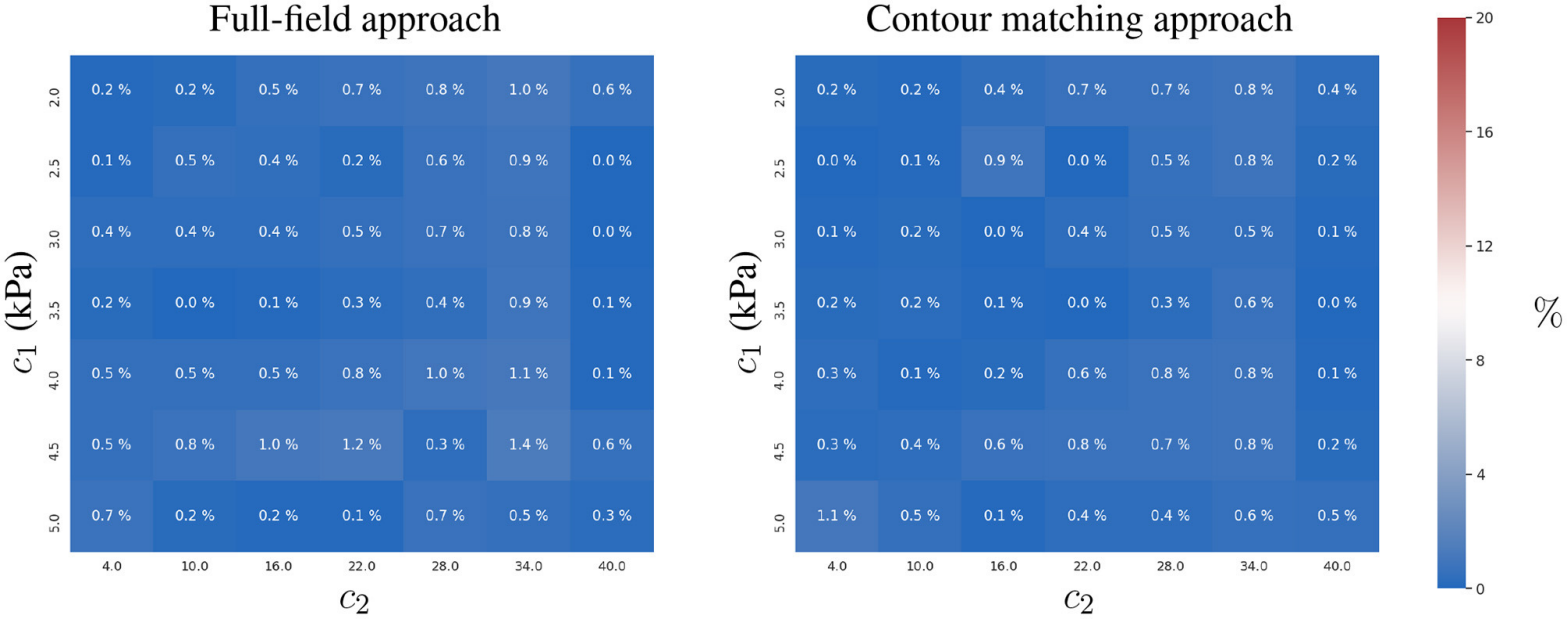
Relative error in estimating intra-ventricular pressure *p* using full-field tracking and contour matching approaches with a load of *p* = 0.9 kPa in the absence of noise in the observations. For details of the estimation of other loads, refer to the Supplementary Material.

Both approaches estimated intra-ventricular pressure *p* with a similar accuracy (relative error ≤ 1%, see Figure 6). The results did not present evidence of a correlation between the magnitude of the displacements and the error in the estimation of *p*.

#### 3.2.2. Experiment 2

**Figure 7:**
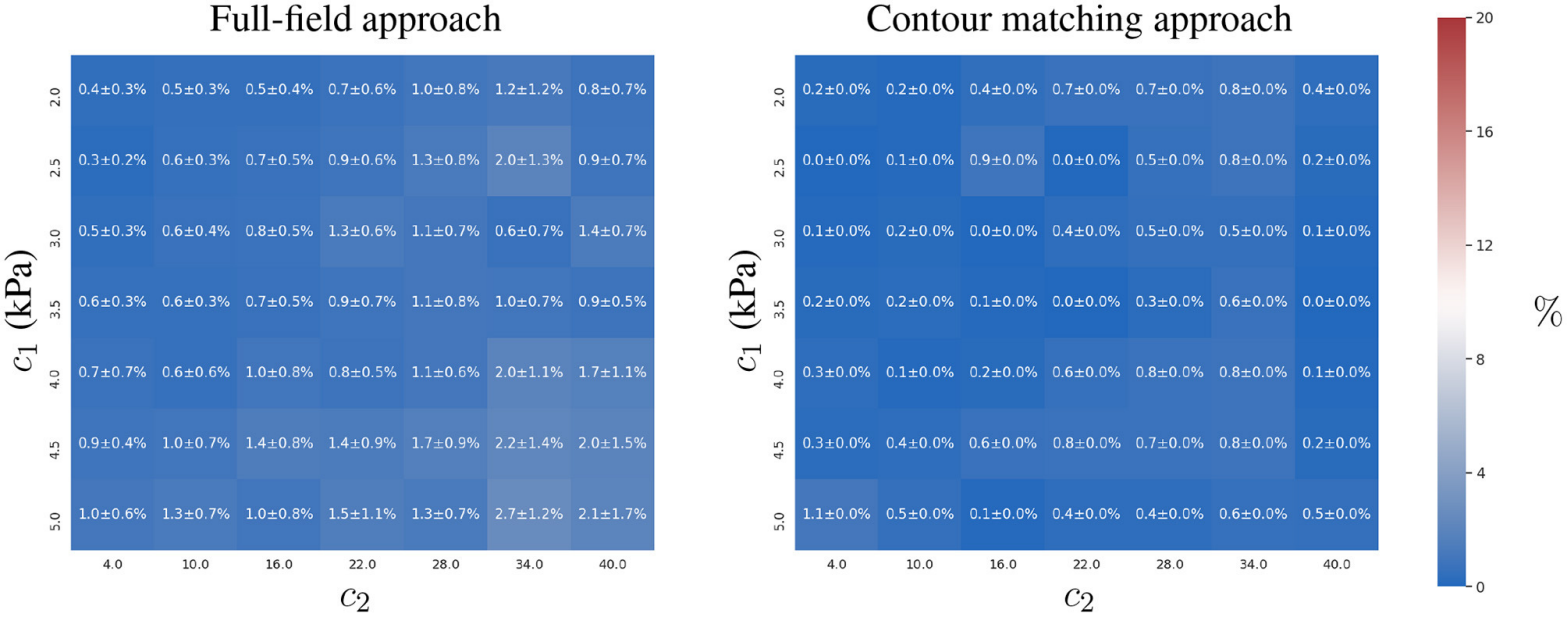
Mean and standard deviation of the relative errors for recovering ground truth intra-ventricular pressure using the full-field tracking and contour matching approaches and an intra-ventricular pressure of *p* = 0.9 kPa with Gaussian noise (σ = 1 mm ≈ 2 pixels) in the observations. Refer to the Supplementary Material for details of the relative errors for other intra-ventricular pressures.

Introducing observation errors slightly reduced the accuracy of the full-field tracking approach (see Figure 7), especially for stiffer materials. The interpretation of these results is analogous to the analysis presented for the estimation of *c*_1_ in Experiment 2 of section 3.1. As reported in the previous section, with increasing material stiffness, the displacements of the ventricle for the same intra-ventricular pressure are smaller and the same intensity of noise will have more detrimental effects on the estimation process (due to less displacement-to-**u**_ϵ_ ratio in the observations).

### 3.3. Convexity of the Objective Function

Both approaches are based on measuring the displacement error by means of the Euclidean distance between the ground truth and the AI-surrogate prediction. As the mechanical model monotonically increases the ventricular displacements with the increase of *p* and the decrease of *c*_1_ and *c*_2_, the minimisation problem defined in Equations (3) or (4) with respect to *p*, *c*_1_ or *c*_2_ is convex if the AI-surrogate *sufficiently* approximates the FE model response.

**Figure 8:**
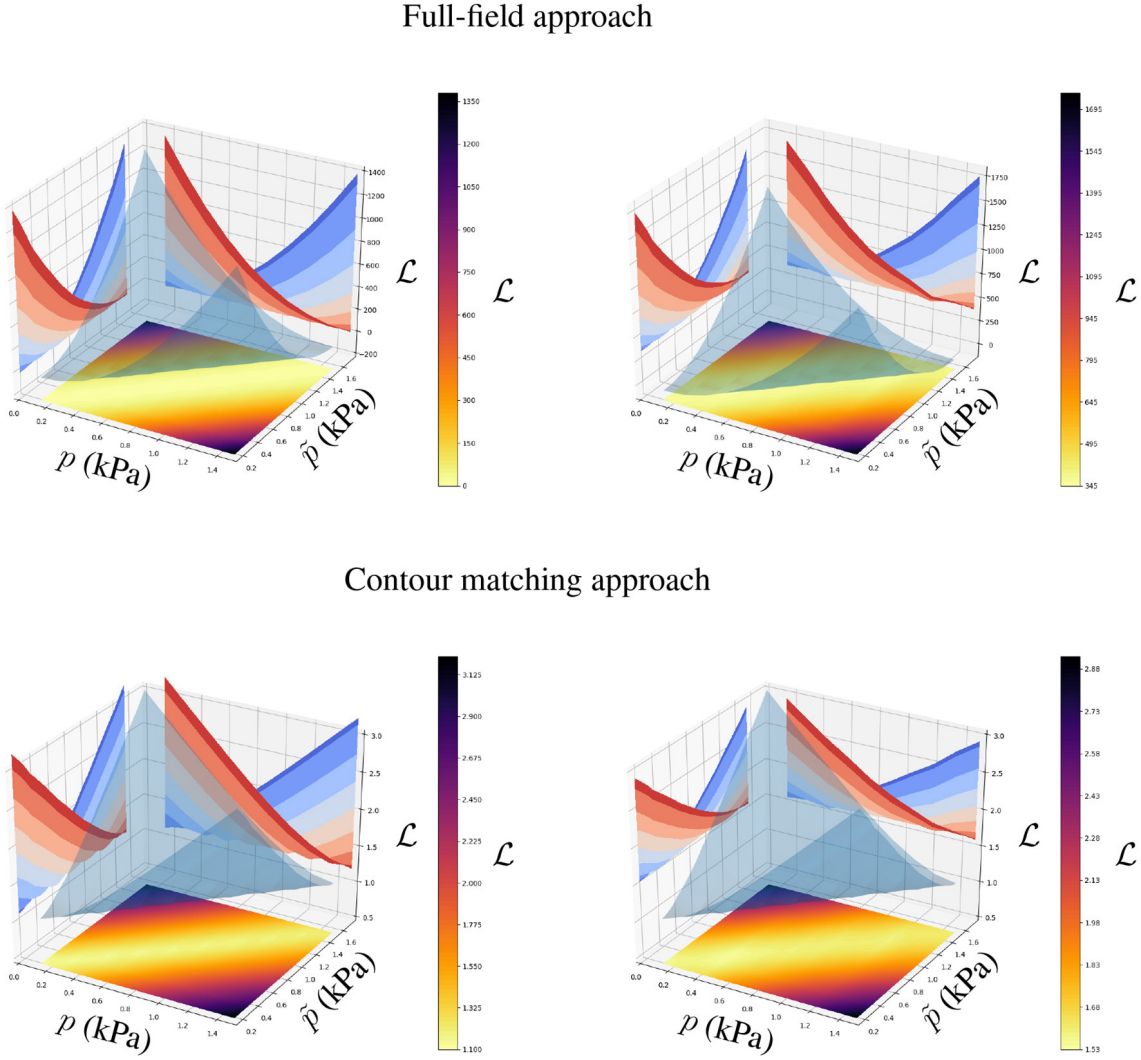
Loss function for the full-field tracking and contour matching approaches with respect to *p* in the presence **(right)** and absence **(left)** of noise in the observations. The surface plot in the *p*-$\hat{p}$ plane and the opaque manifold represent the loss function value L(p) when $\hat{p}$ is the ground truth (optimal) value. Level curves in L-$\hat{p}$ and L-*p* planes correspond to parallel cuts of the opaque manifold for fixed values of *p* and $\hat{p}$, respectively (blue to red shades indicate values of *p* and $\hat{p}$ that correspond to the ticks on their respective axes). Note that for a given $\hat{p}$, L(p) is convex with the minimum $p=\hat{p}$ resulting in precise and well-behaved formulations for gradient-based optimisers.

**Figure 9:**
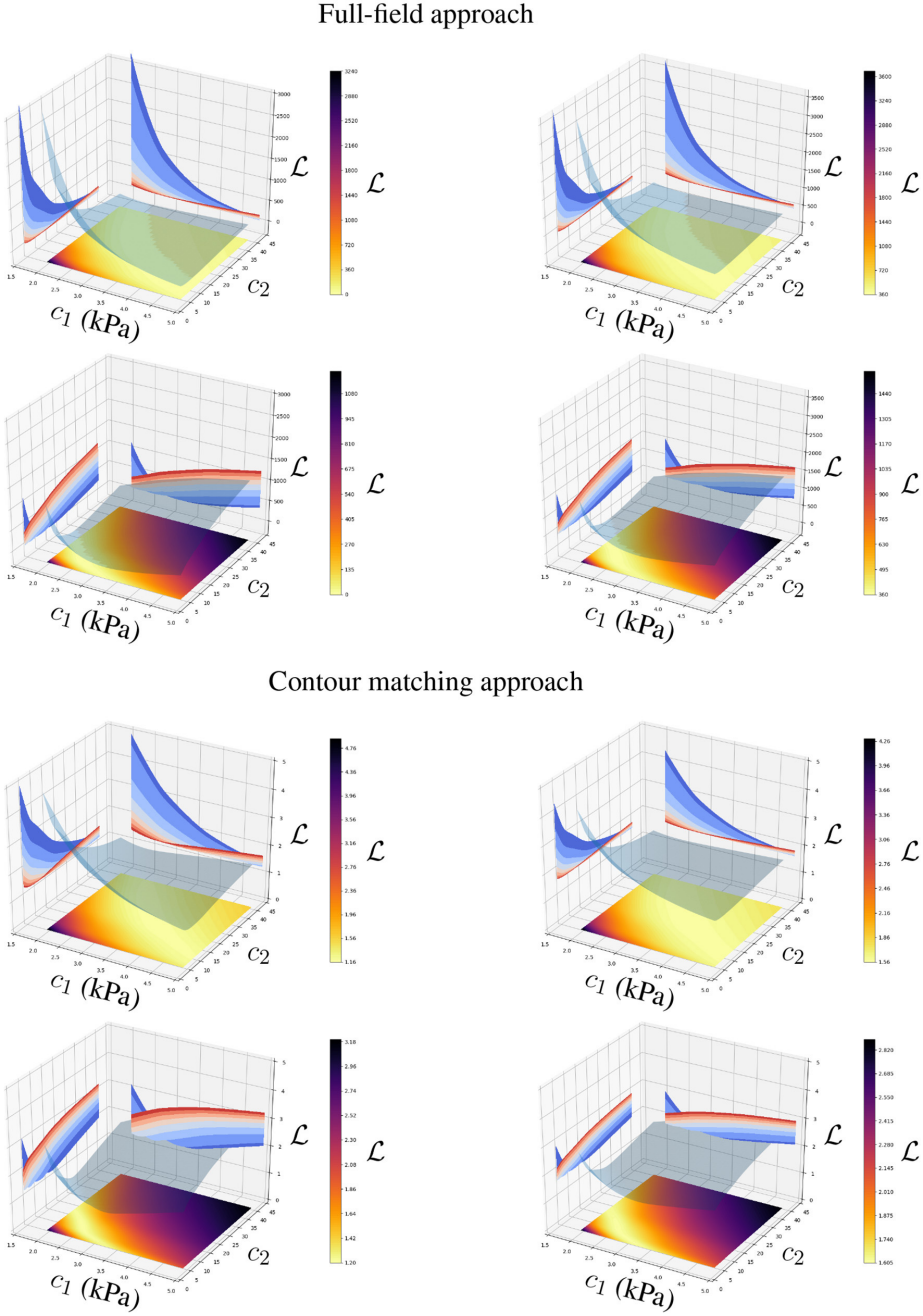
Loss function for the full-field tracking and contour matching approaches with respect to *c*_1_ and *c*_2_ in the presence **(right)** and absence **(left)** of noise in the observations. The first and second rows present the loss function values when *c*_1_ = 3.5 kPa, *c*_2_ = 22.0 and *c*_1_ = 2.5 kPa, *c*_2_ = 10.0 are the ground truth parameters, respectively. Surface plots in *c*_1_-*c*_2_ plane and the opaque manifolds represent the loss function value $L\left(c_{1},c_{2}\right)$. Level curves in L-*c*_1_ and L-*c*_2_ planes correspond to parallel cuts of the opaque manifold for fixed values of *c*_2_ and *c*_1_, respectively (blue to red shades indicate values of *c*_1_ and *c*_2_ that correspond to the ticks on their respective axes). Note that the objective functions are more sensitive to perturbations of *c*_1_ and *c*_2_ for softer materials (as highlighted by the blue level curves, which represent lower values of *c*_1_ and *c*_2_).

The AI-surrogate used in this manuscript, satisfies such a condition (see Figures 8, 9). We can observe convexity in the loss function with respect to *p* and the constitutive parameters *c*_1_ and *c*_2_. Because we use a stochastic gradient descent optimiser, the identifiability of the parameters is related to the rate of change of the loss function with respect to the parameter of interest. We observed a decrease in $\frac{\partial L}{\partial c_{1}}$ and $\frac{\partial L}{\partial c_{2}}$ for stiffer materials. Nonetheless, both approaches presented good accuracy in recovering physiologically realistic ranges of parameters (2.0 kPa ≤ *c*_1_ ≤ 5.0 kPa and 4.0 ≤ *c*_2_ ≤ 40.0, even in the presence of noise (see Figures 8, 9). The identifiability of *p* does not exhibit degradation in the physiological range, even in the presence of noise (see Figure 8).

### 3.4. Computational Cost

We quantify the performance of the formulations by measuring the wall-clock time taken to estimate four different set of parameters: (i) intra-ventricular pressure; (ii) constitutive parameter *c*_1_; (iii) constitutive parameter *c*_2_; and (iv) constitutive parameters *c*_1_ and *c*_2_. For each test, we executed the estimation 50 times with different observation errors (using the same noise distribution presented in section 3). During these tests, the observation $\hat{\text{u}}$ corresponded to a ground truth FE model prediction obtained using *p* = 0.9 kPa, *c*_1_ = 3.5 kPa and *c*_2_ = 22.0. We then estimated the mean and standard deviation of the wall-clock times over the 50 executions (see Table 1). The networks and optimisation schemes were implemented in TensorFlow 2.1 with GPU-support, using an NVIDIA Quadro P6000 and CUDA v11.0 for their executions.

**Table 1:** Wall-clock times for parameter estimation using the proposed strategies.

**Approach**	**Estimated parameters**			
	**p (s)**	***c*_1_ (s)**	***c*_2_ (s)**	***c*_1_, *c*_2_ (s)**
Full-field tracking	1.0 ± 0.3	1.2 ± 0.3	1.2 ± 0.3	5.0 ± 1.6
Contour matching	6.1 ± 1.2	8.6 ± 0.8	7.7 ± 1.0	17.7 ± 1.0

*The convergence criterion introduced in Equation (7) was used for all cases. The observation data corresponds to the FE model prediction using parameters p = 0.9 kPa, c_1_ = 3.5 kPa and c_2_ = 22.0*.

Both approaches were able to estimate the different set of parameters in less than 18 s. In particular, the full-field tracking approach executed 6.2, 7.3, 6.4, and 3.6 times faster for experiments (i)-(iv), respectively, than the contour matching counterpart. The slower response of the contour matching is due to the oversampling of the ventricular surface ${\text{s}}_{f}^{\cdot }$ that is necessary for obtaining a good approximation of Equation (5). Such oversampling increases the number of points in **s**_2_ requiring more model evaluations and thus computational expense.

In terms of computational complexity, the full-field tracking approach has a cost of $O\left(\left|\hat{\text{u}}\right|\right)$ where $\left|\hat{\text{u}}\right|$ is the number of points across the ventricular wall, and the contour matching approach has a cost of $O\left(\left|{\text{s}}_{1}||{\text{s}}_{2}\right|\right)$ where |**s**_*i*_| is the number of points at the ventricular surfaces **s**_*i*_. This analysis shows a higher computational complexity for the contour matching approach, which detrimentally impacts the scaling of the approach with respect to the discretisation of the ventricle. Specifically, the computational cost grows linearly and quadratically for the full-field tracking and contour matching approaches, respectively.

Another contribution to the lower performance of the contour matching approach is given by the number of network evaluations. The total number of evaluations of $\text{u}\left(\hat{\Theta }\right)$ scales linearly with respect to the discretisation of the ventricle for both formulations. However, contour matching requires almost twice the number of evaluations when compared to the full-field tracking approach (1109 evaluations for each contour, vs. 1072 evaluations for tracking). Thus, less refined representations of the ventricular surfaces may help reduce the computational expense if needed for clinical translation.

It is worth noting that we optimised Equations (3) and (4) using the ADAM algorithm, because of its direct support toward neural network optimisation. It is possible that the use of efficient optimisers for convex problems, such as L-BFGS (Liu and Nocedal, 1989) or even Newton's method, may further enhance convergence and reduce the computational effort in the proposed formulations.

## 4. Discussion

The two techniques proposed for parameter estimation feature appealing properties such as low computational cost, simple implementation, and no need for analytical derivatives of the objective function. To achieve this, we used automatic differentiation, already implemented in neural networks frameworks (such as Tensorflow and PyTorch), for solving Equations (3) and (4). This allow us to use exact gradient information from complex AI-surrogate models without any additional effort. Thus, we were able to assess the sensitivity of the objective function residuals with respect to its inputs, allowing for an efficient convex optimisation of the inputs. Additionally, the same neural network frameworks are endowed with GPU-efficient implementations, accelerating the evaluation of models by orders of magnitude with respect to a cost equivalent CPU infrastructure.

Regarding the estimation of the intra-ventricular pressure and constitutive parameters of the left ventricle, we conclude that both of the proposed approaches can provide accurate predictions of parameters, even in the presence of measurement noise. The noise was modelled as a normal zero-mean distribution with a standard deviation of two pixels. This noise represented reconstruction errors of the ventricle wall displacement (full-field tracking approach), or the ventricular surface geometry (contour matching approach). Note that in our analysis, the errors present no biases. If a bias were present (e.g., consistent segmentation errors due to mis-identification of the structures, or assimilating experimental data with a model with a significant modelling error), this may lead to larger estimation errors than those reported here.

Both approaches demonstrated good identification properties for the physiological range considered in our experiments. Particularly, we focused on the analysis of relatively stiffer materials (*c*_1_ > 2.0 kPa) and lower intraventricular pressures (*p* = 0.9 kPa) because it present a more challenging scenario to assess the parameter estimation task. As displacements are smaller for lower pressures and stiffer materials, the noise and the displacement field are within the same displacement magnitudes, and a lower performance for the estimation is expected. However, the results only showed this degradation under those conditions for the full-field tracking approach, increasing its error estimate from 0.6 to 1.8% in *c*_1_, from 1.9 to 4.6% in *c*_2_ and from 0.3 to 2.7% in *p* (see Figures 5, 7). The contour matching approach only presented a slight degradation in parameter estimates of *c*_2_ when *c*_1_ reached the upper bound of its physiological range. Note that, after estimating the constitutive parameters, the displacement errors between the AI-surrogate predictions and the target observations were visually negligible when the corresponding ventricle contours were overlaid. The case with the largest disagreement across all simulations reported here is shown in Figure 4 for the full-field approach with target parameters *c*_1_ = 3.5 kPa and *c*_2_ = 10.0 (the specific sample estimated *c*_1_ = 3.76 kPa and *c*_2_ = 7.05). In this case, the displacements of the AI-surrogate had an error of 0.06 ± 0.02 mm with respect to the FE ground truth. In particular, when not using information from multiple frames, the full-field tracking approach failed to precisely identify *c*_2_ with errors reaching 16 ± 9.6%. Nonetheless, in practical applications, clinical MR datasets often contain enough temporal resolution to perform a multi-frame kinematic assessment.

For normal human physiological cases (diastasis pressures of approximately 1.2 kPa), both methods present a slight improvement in performance due to the larger displacements in the observations (see Supplementary Figures 3, 7, 11, 15). As the observed displacements increase, the approximation error of the AI-surrogate to the FE model has a smaller contribution, leading to more accurate estimates. Specifically, we can observe this for the estimates of stiffer materials, where the larger displacements improved the parameter identification in comparison with the 0.9 kPa pressure case.

Regarding clinical translation, this approach offers rapid and efficient estimation of the mechanical properties using commodity computational resources, e.g., a standard computer with state-of-the-art GPU. The generated training datasets used in section 3 assume realistic data constraints (i.e., observation errors and resolution) expected in medical data. In this study, we assumed segmentation errors of two pixels, and a temporal resolution of 10 frames during diastole (see section 3), which are both attainable using a 3T clinical MRI scanner. FE models (which are used to train our AI-surrogate) have demonstrated clinical utility (Wang et al., 2018; Hasaballa et al., 2021), evidencing the suitability of this approach for mechanical characterisation of the heart. Nonetheless, robust uncertainty quantification analysis should be performed to analyse all sources of error in the specific clinical environment. For the assessment of mechanical properties of the ventricular wall, it is important to quantify the uncertainty in the pressure measurements, segmentation error, and geometric representation error. The characterisation of such uncertainties is out of the scope of the present work.

In terms of the number of parameters to be estimated, our study demonstrated reasonable efficiency for the simultaneous estimation of two parameters. Compared to the single parameter problem, the computational time for the two parameter problem was more than two-fold, but still presenting time ranges compatible with clinical practice. This is due to the coupled effect of the parameters in the model response which is a problem shared by all parameter estimation techniques. The extension of our technique for the simultaneous identification of additional parameters is possible, as long as the objective function remains strictly convex with respect to the parameters of interest.

While the estimation approaches considered in this work involved solving convex optimisation problems, the use of AI-surrogates can offer advantages for non-convex problems. In such cases, non-convex optimisation solvers, such as genetic algorithms, may benefit from the use of AI-surrogates due to the computationally inexpensive model evaluations. This feature enables a more efficient exploration of the parameter space with reduced computational intensity.

Finally, both of the proposed estimation techniques are general, and can be translated straightforwardly to other applications (e.g., to estimate constitutive properties of other tissues, such as the breast, lung or liver) as long as an AI-surrogate can be generated from the appropriate models. The full-field tracking approach is limited to applications where material point tracking measurements are available (e.g., using CMR tissue tagging, or image registration techniques). On the other hand, the contour matching approach can be applied to any applications where the surfaces of the tissues or organs of interest can be quantified experimentally.

## 5. Conclusions

This study proposed two approaches for parameter estimation using AI-surrogates, depending on whether (i) tracking kinematic measurements, or (ii) only surface measurements are available. We focused our application on the estimation of left ventricular constitutive properties and its intra-ventricular pressure during the passive filling phase of the cardiac cycle.

We conclude that: (i) both approaches are robust with respect to Gaussian noise when the measurement data for multiple loading conditions were combined; and (ii) estimates of one or two constitutive parameters could be obtained in less than 9 or 18 s, respectively. We found that the contour matching approach was more robust toward Gaussian noise, recovering the ground truth parameters with high accuracy even when only one loaded configuration was available. Conversely, the full-field tracking approach was more efficient than its counterpart by a factor of ≈4, while providing the possibility of further improving scalability as medical imaging resolution improves.

## Data Availability Statement

The data analyzed in this study is subject to the following licenses/restrictions: The de-identified data is available from the authors upon reasonable request, with permission of the institutional review board and under ethical approval. Requests to access these datasets should be directed to Martyn P. Nash, martyn.nash@auckland.ac.nz.

## Author Contributions

GM co-designed the study, implemented, validated and executed the optimisation techniques, and prepared the manuscript. TB co-designed the study, developed the biomechanical modelling framework, and contributed to the manuscript text. MN contributed to the design of the study, the development of the biomechanical modelling framework, and edited the manuscript. All authors contributed to the discussions presented in this manuscript.

## Funding

This work was supported by research funding grants 13/317 and 17/608 from the Health Research Council of New Zealand, and funding grant 9077/31/8402 from the Li Ka Shing Foundation.

## Conflict of Interest

GM and TB are scientific advisors at HeartLab (NZ) Ltd. MN is Chief Scientific Officer at HeartLab (NZ) Ltd.

## Publisher's Note

All claims expressed in this article are solely those of the authors and do not necessarily represent those of their affiliated organizations, or those of the publisher, the editors and the reviewers. Any product that may be evaluated in this article, or claim that may be made by its manufacturer, is not guaranteed or endorsed by the publisher.
