# Supplementary material for: Efficient Ventricular Parameter Estimation Using AI-Surrogate Models
Source: Front Physiol. 2021 Oct 14;12:732351. doi: 10.3389/fphys.2021.732351 (PMC8551833; doi:10.3389/fphys.2021.732351)
Supplement: Supplementary file 1 [file Data_Sheet_1.PDF]

frontiers

Supplementary Material

As follows, we report additional results obtained from Experiments 1 and 2 in Sections 3.1 and 3.2 using different intra-ventricular pressure within the physiological range of loads (0.3 kPa, 0.6 kPa,1.2 kPa and 1.5 kPa - see Figures S1-S16). Particularly, $p = 1.5$ kPa is associated with the end of the diastole (end of the filling stage when the mitral valve closes), and yields the largest displacements from diastasis. Considering that observation errors do not scale with respect to the observed displacements –which is reasonable as no motion artefacts are present through the filling phase in MR acquisition–, the estimation of the parameters with a two-frames setup is potentially the most reliable using diastasis and end-diastolic frames to generate the observations.

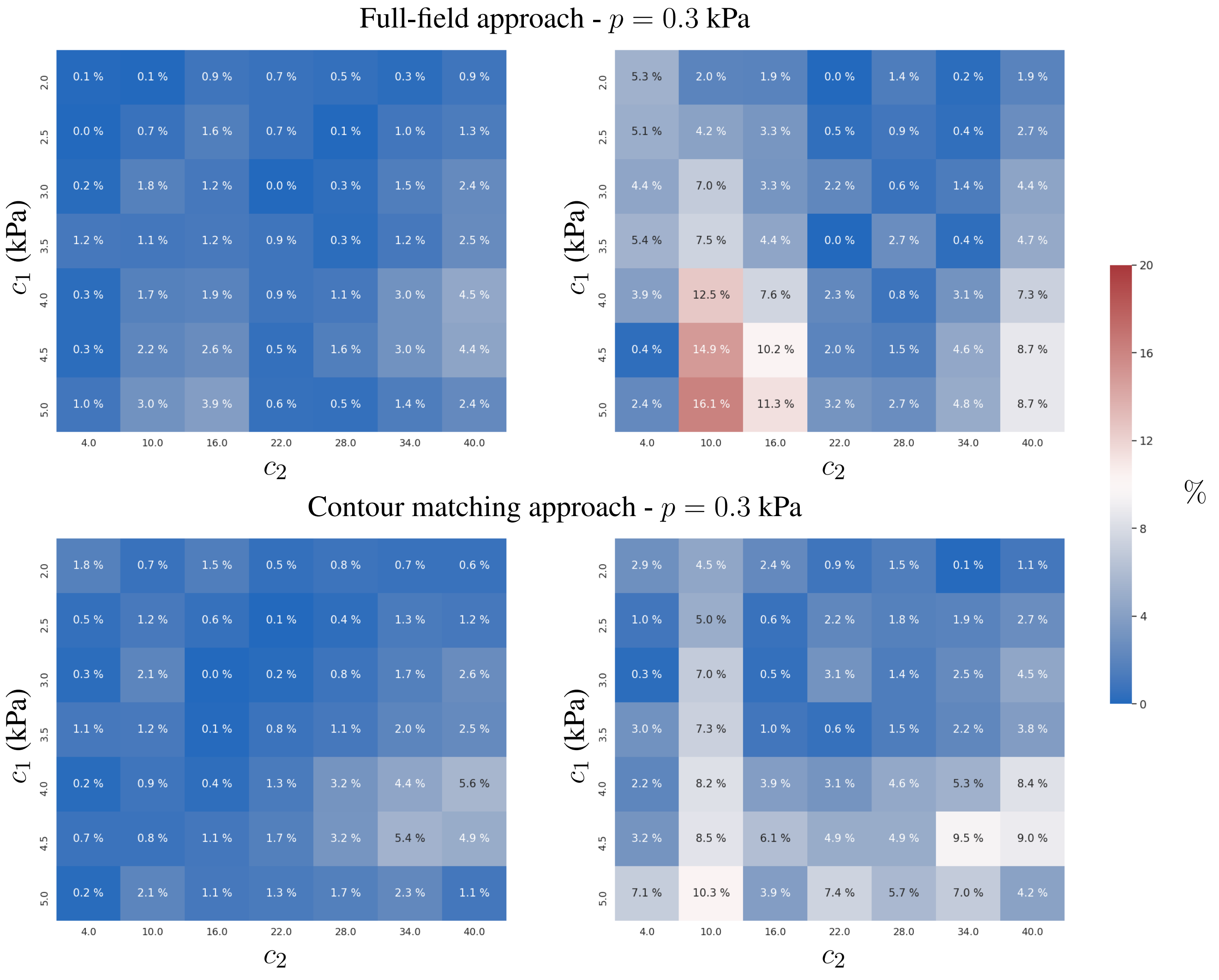

Figure S1. Estimates of relative error for c_1 (left) and c_2 (right) constitutive parameters using the full-field tracking and contour matching approaches with a load of $p = 0.3$ kPa in the absence of noise in the observations.

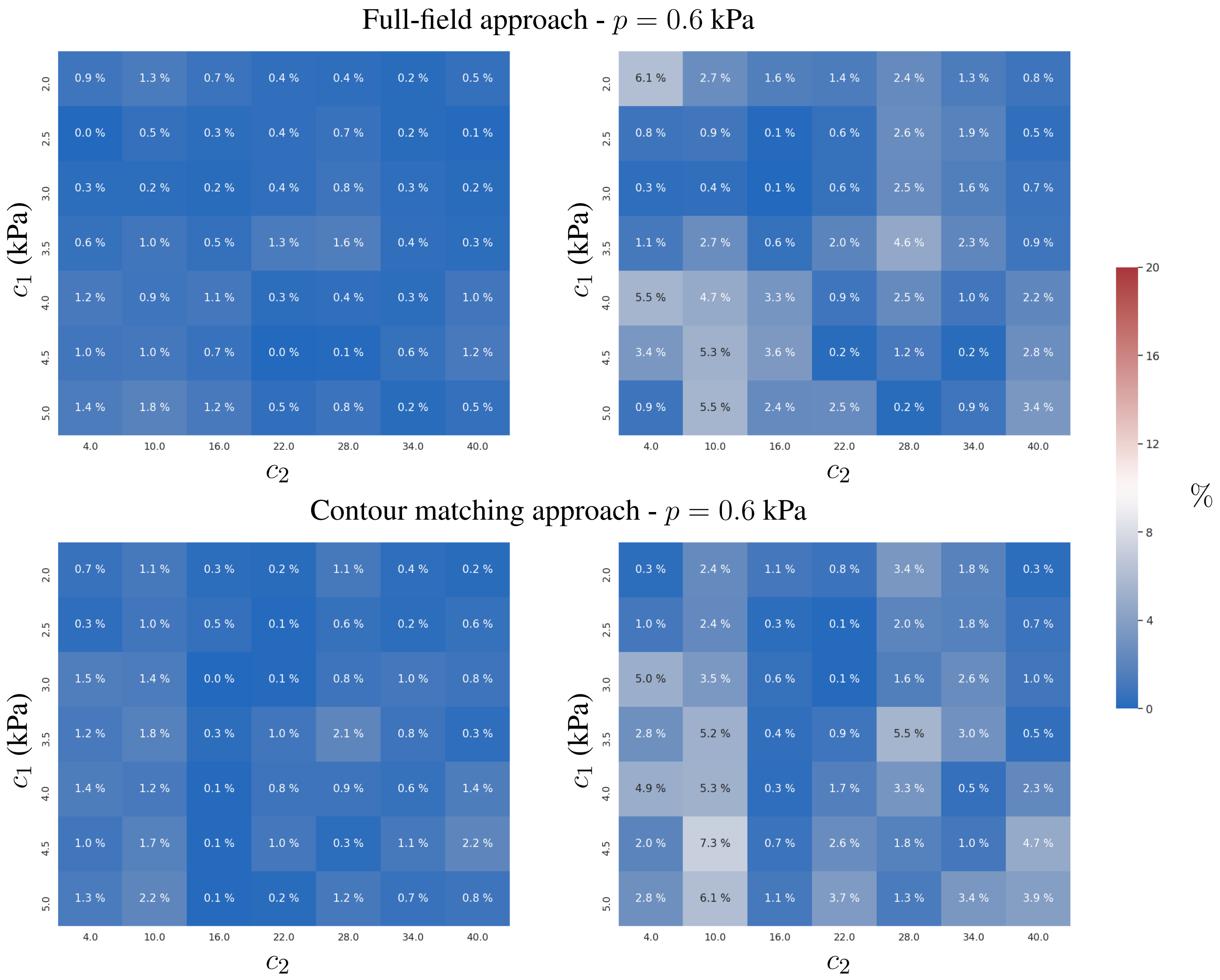

Figure S2. Estimates of relative error for c_1 (left) and c_2 (right) constitutive parameters using the full-field tracking and contour matching approaches with a load of $p = 0.6$ kPa in the absence of noise in the observations.

Additionally, we report the mean and standard deviation of the displacements for different constitutive materials and intra-ventricular pressures to characterise the magnitude of the displacements expected in the different experiments (see Figures S17-S20).

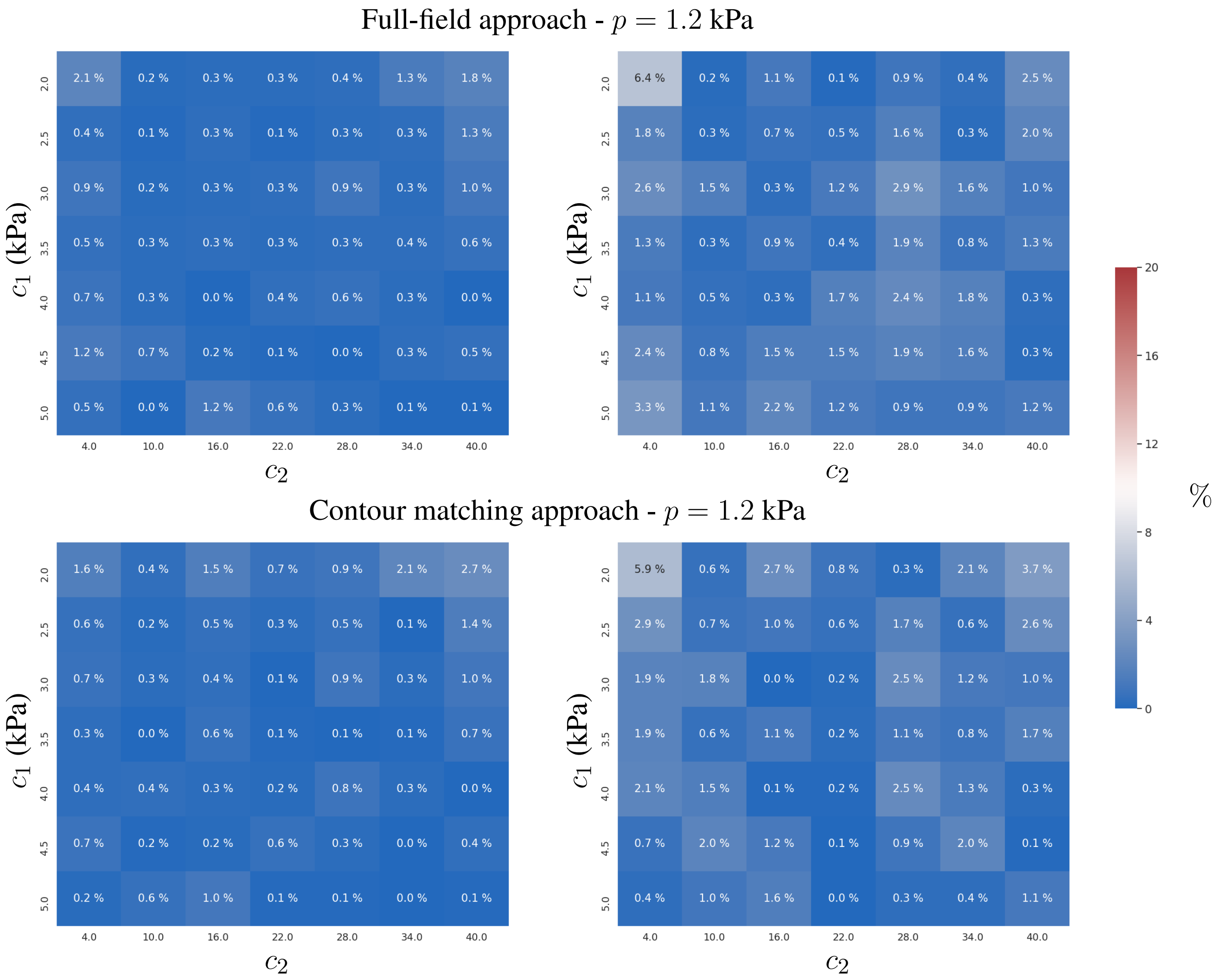

Figure S3. Estimates of relative error for c_1 (left) and c_2 (right) constitutive parameters using the full-field tracking and contour matching approaches with a load of $p = 1.2$ kPa in the absence of noise in the observations.

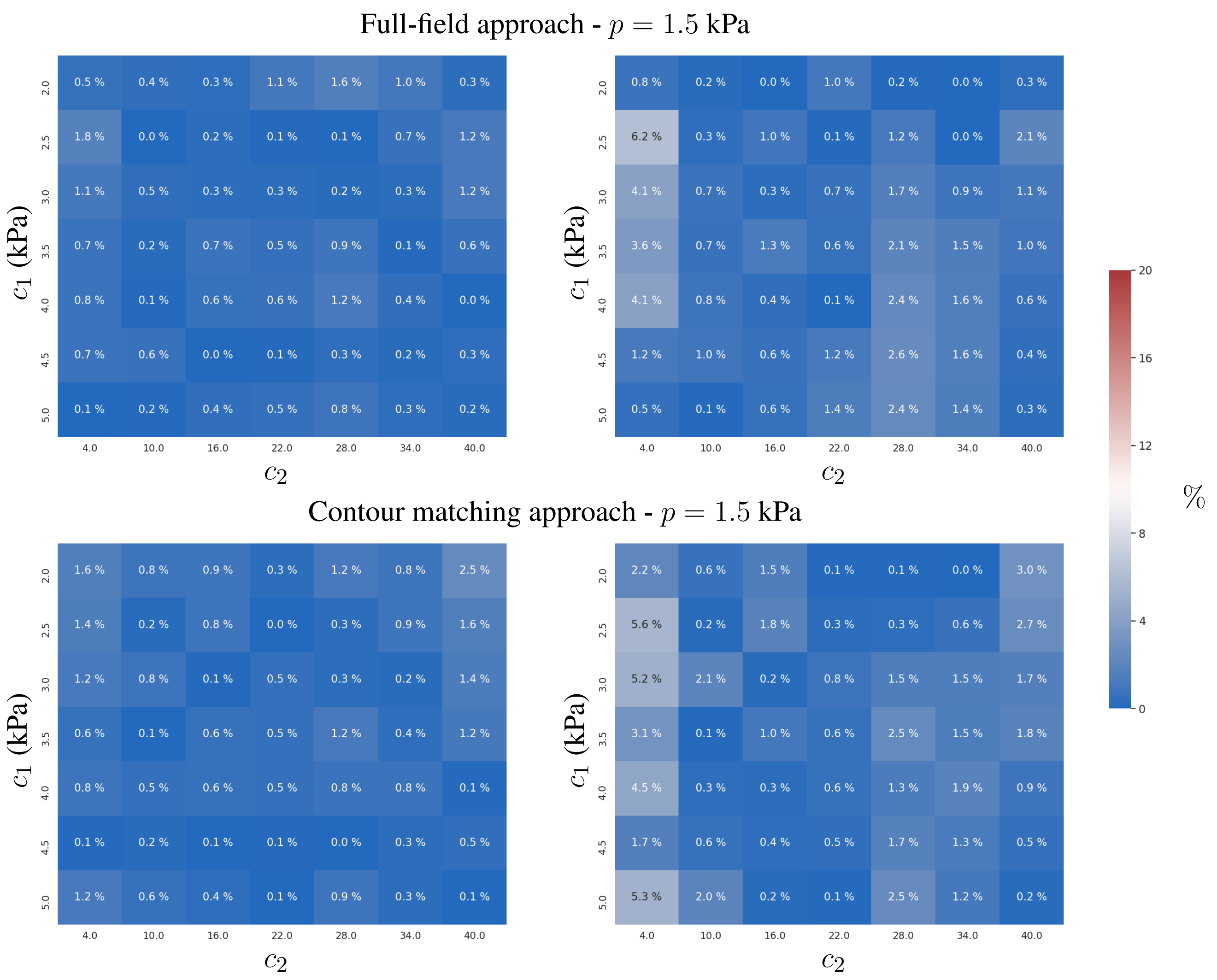

Figure S4. Estimates of relative error for c_1 (left) and c_2 (right) constitutive parameters using the full-field tracking and contour matching approaches with a load of $p = 1.5$ kPa in the absence of noise in the observations.

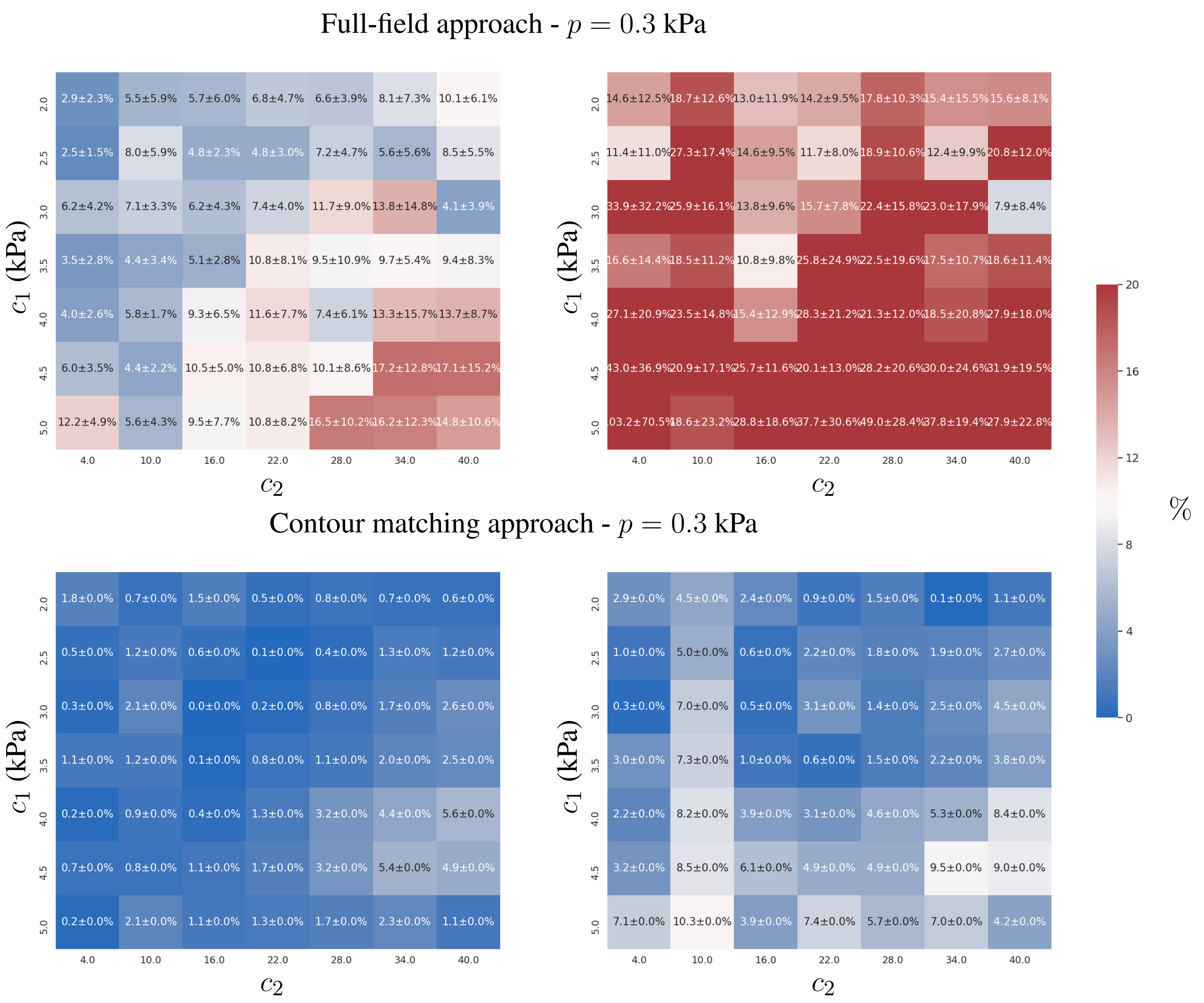

Figure S5. Mean and standard deviation of the relative error for the estimation of c_1 (left) and c_2 (right) constitutive parameters using the full-field tracking and contour matching approaches with a load of $p = 0.3$ kPa and the presence of Gaussian noise ($\sigma = 1\text{mm} \approx 2$ pixels) in the observations.

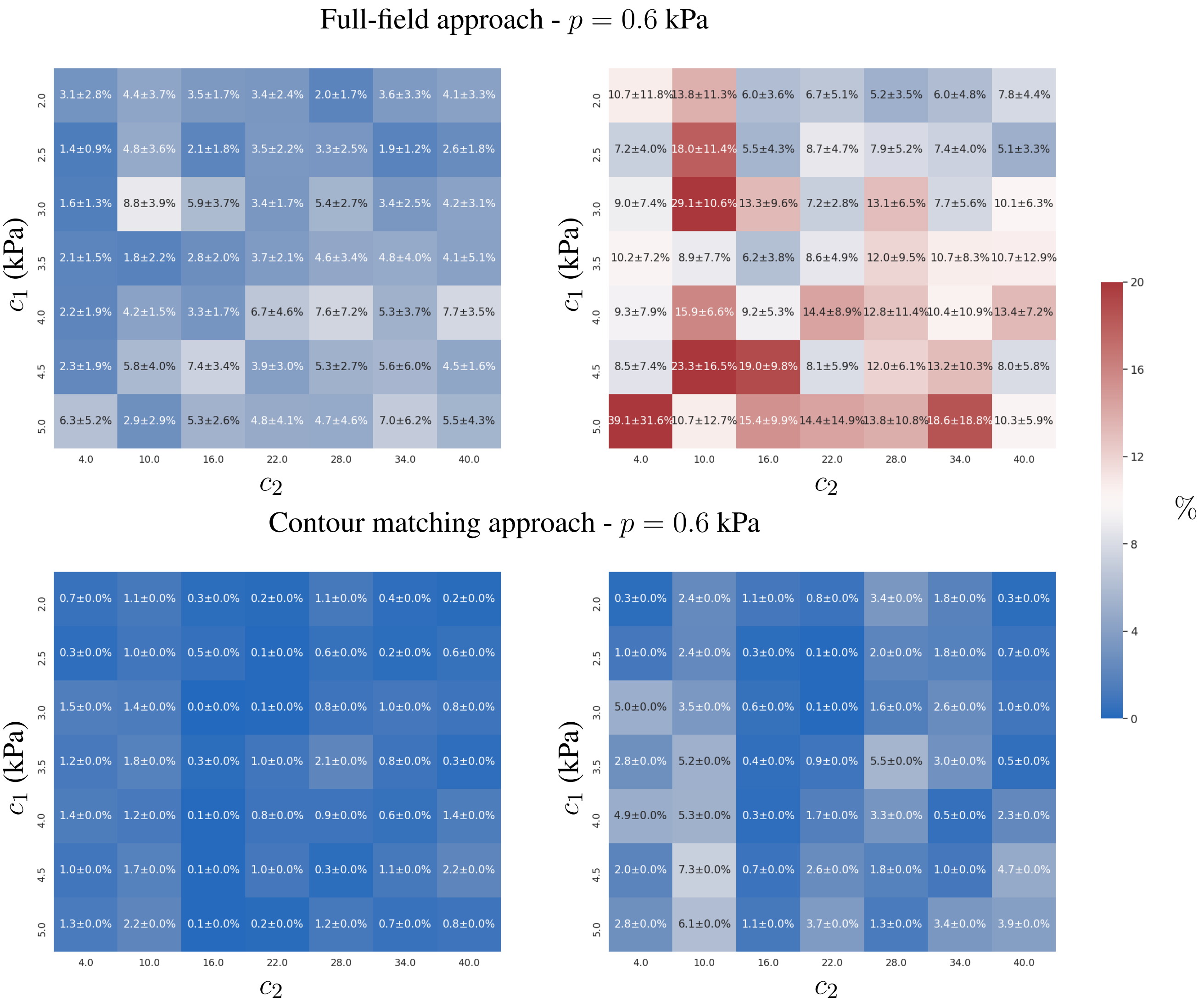

Figure S6. Mean and standard deviation of the relative error for the estimation of c_1 (left) and c_2 (right) constitutive parameters using the full-field tracking and contour matching approaches with a load of $p = 0.6$ kPa and the presence of Gaussian noise ($\sigma = 1\text{mm} \approx 2$ pixels) in the observations.

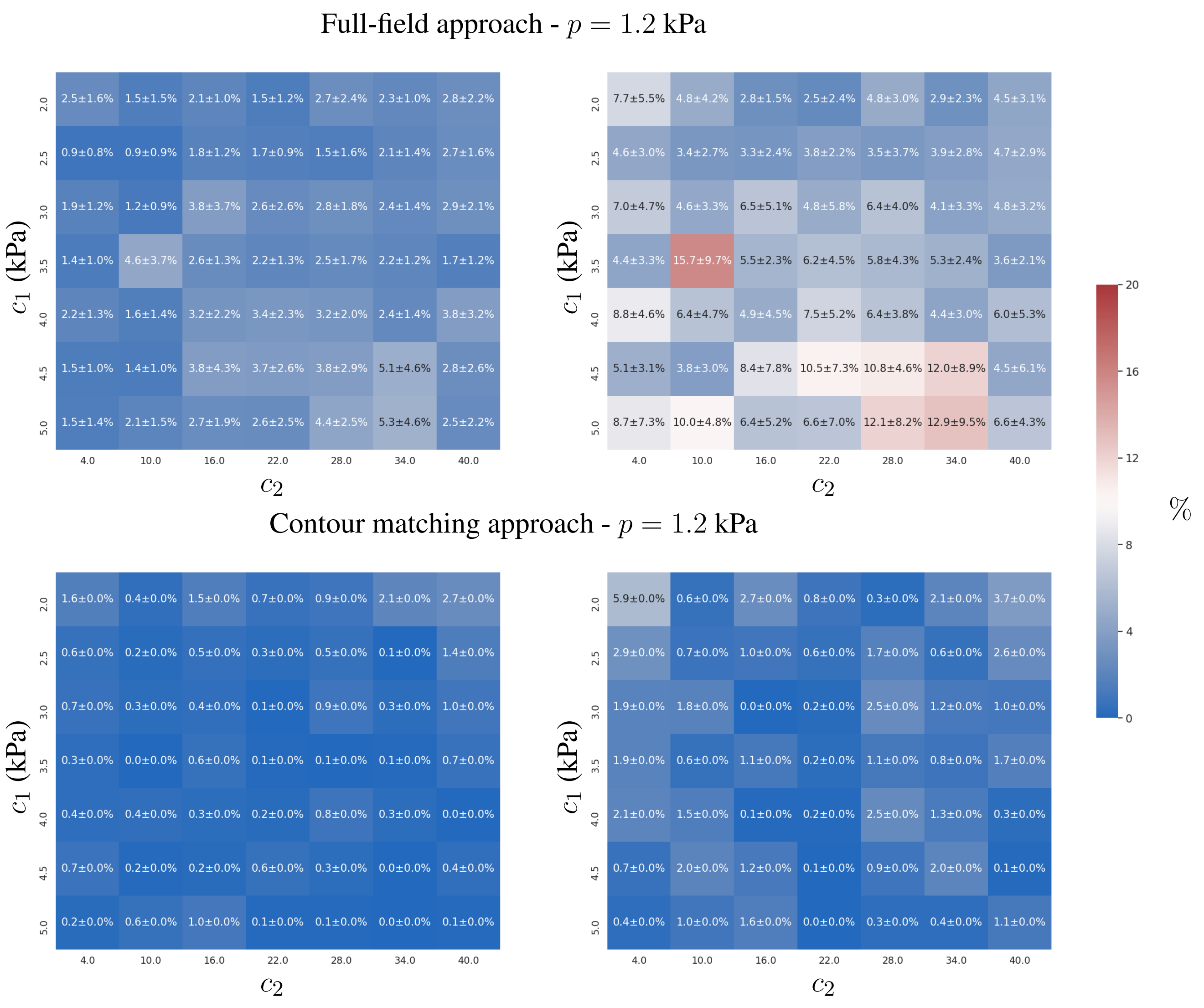

Figure S7. Mean and standard deviation of the relative error for the estimation of c_1 (left) and c_2 (right) constitutive parameters using the full-field tracking and contour matching approaches with a load of $p = 1.2$ kPa and the presence of Gaussian noise ($\sigma = 1\text{mm} \approx 2$ pixels) in the observations.

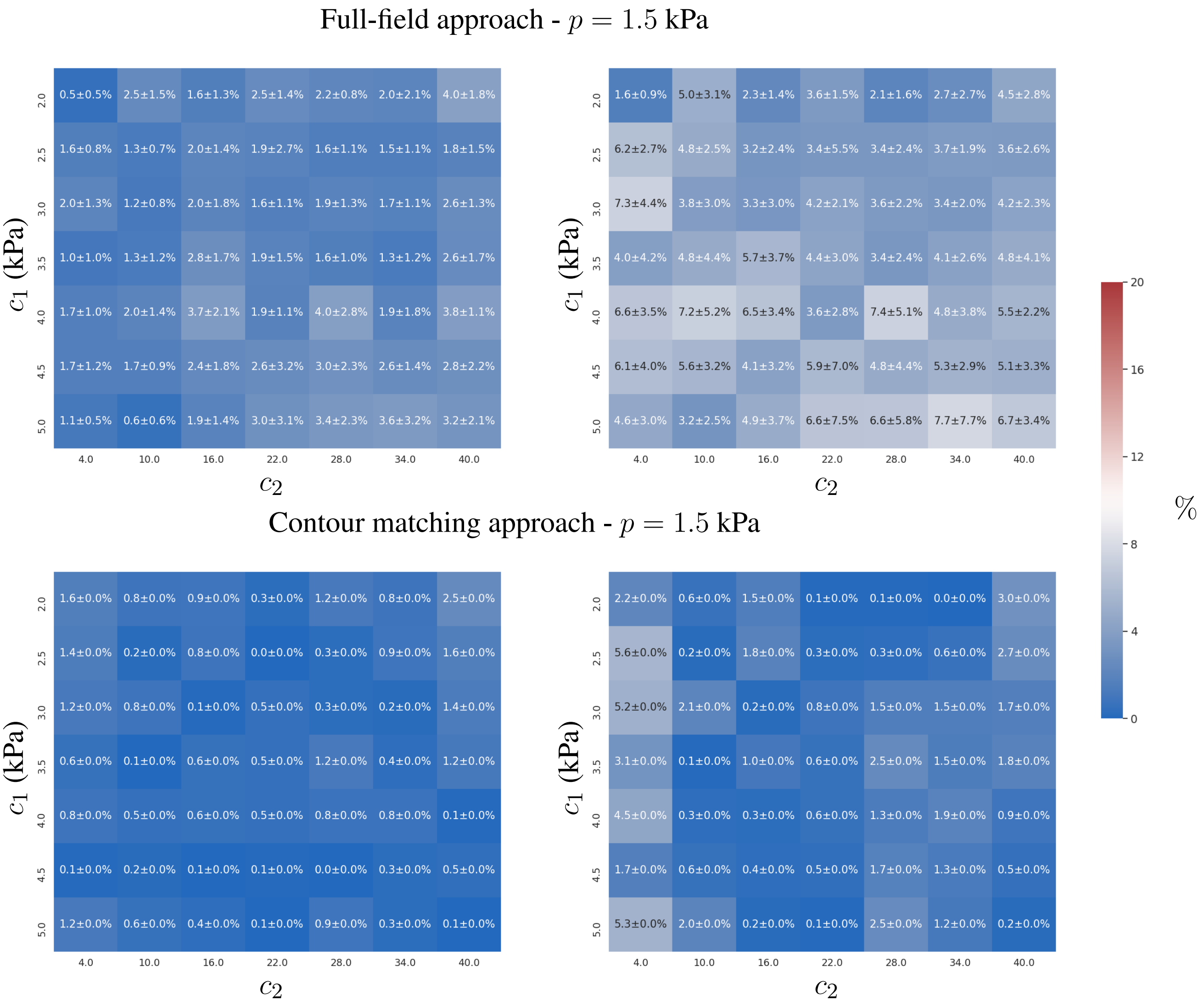

Figure S8. Mean and standard deviation of the relative error for the estimation of c_1 (left) and c_2 (right) constitutive parameters using the full-field tracking and contour matching approaches with a load of $p = 1.5$ kPa and the presence of Gaussian noise ($\sigma = 1$mm ≈ 2 pixels) in the observations.

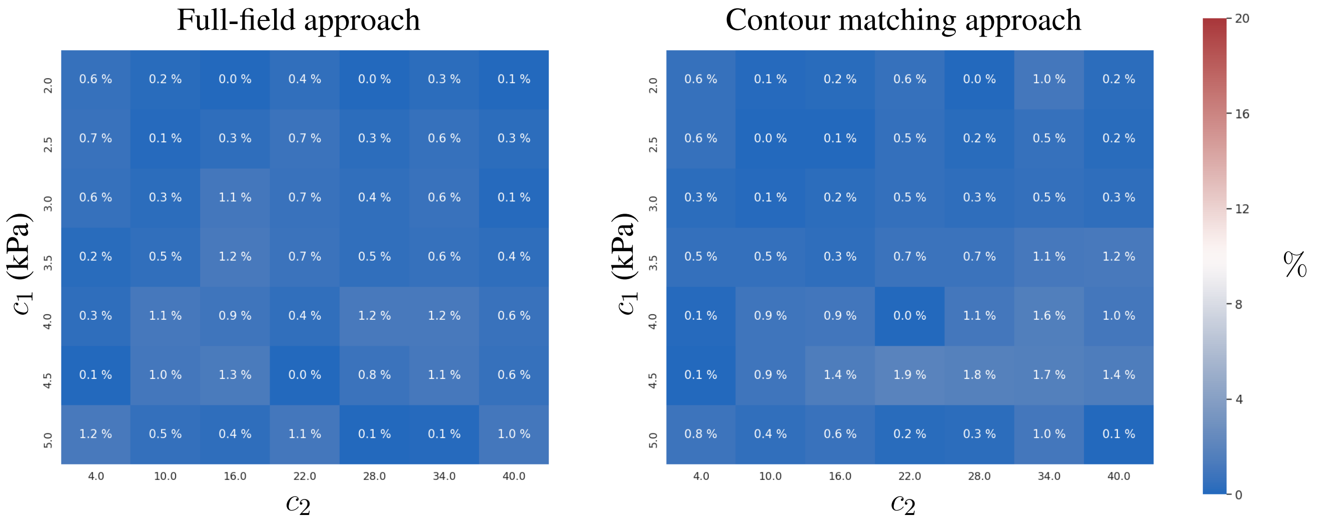

Figure S9. Relative error in estimating intra-ventricular pressure p using full-field tracking and contour matching approaches with a load of $p = 0.3$ kPa in the absence of noise in the observations.

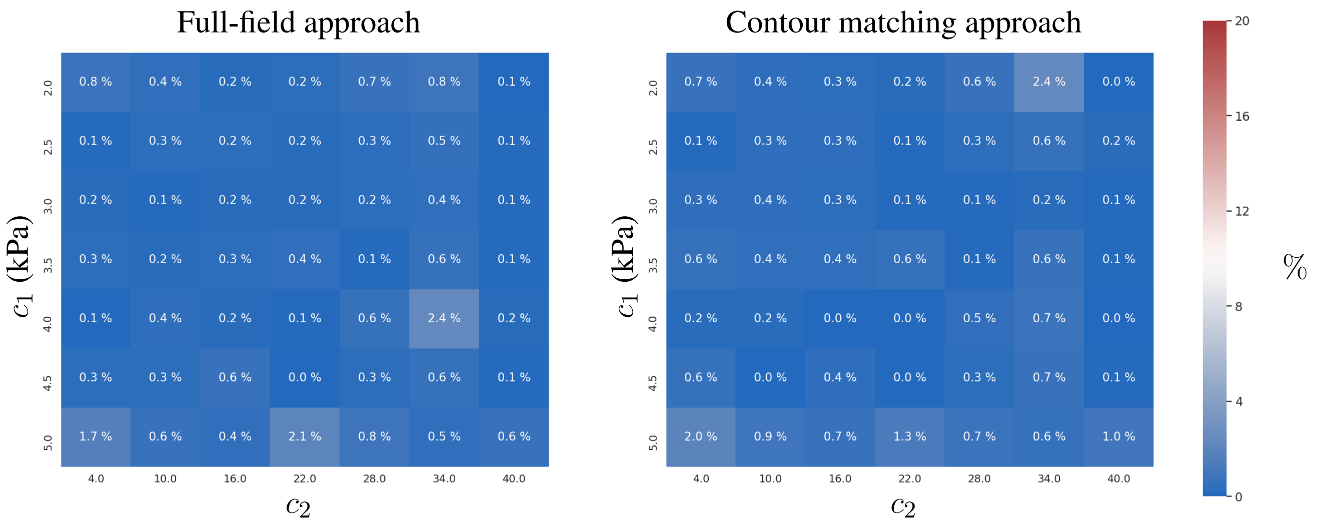

Figure S10. Relative error in estimating intra-ventricular pressure p using full-field tracking and contour matching approaches with a load of $p = 0.6$ kPa in the absence of noise in the observations.

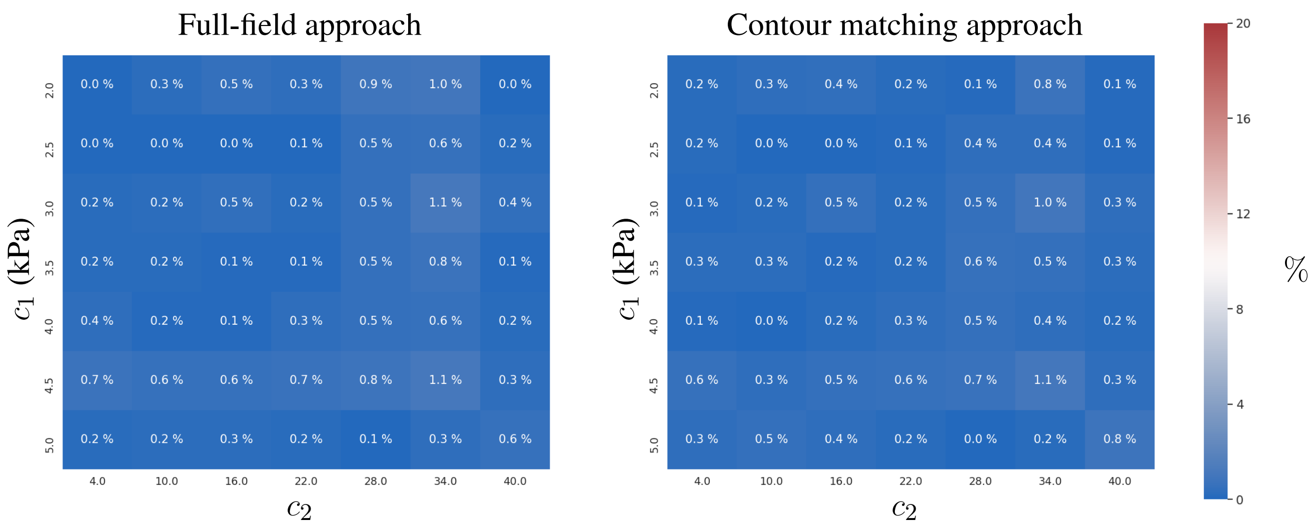

Figure S11. Relative error in estimating intra-ventricular pressure p using full-field tracking and contour matching approaches with a load of $p = 1.2$ kPa in the absence of noise in the observations.

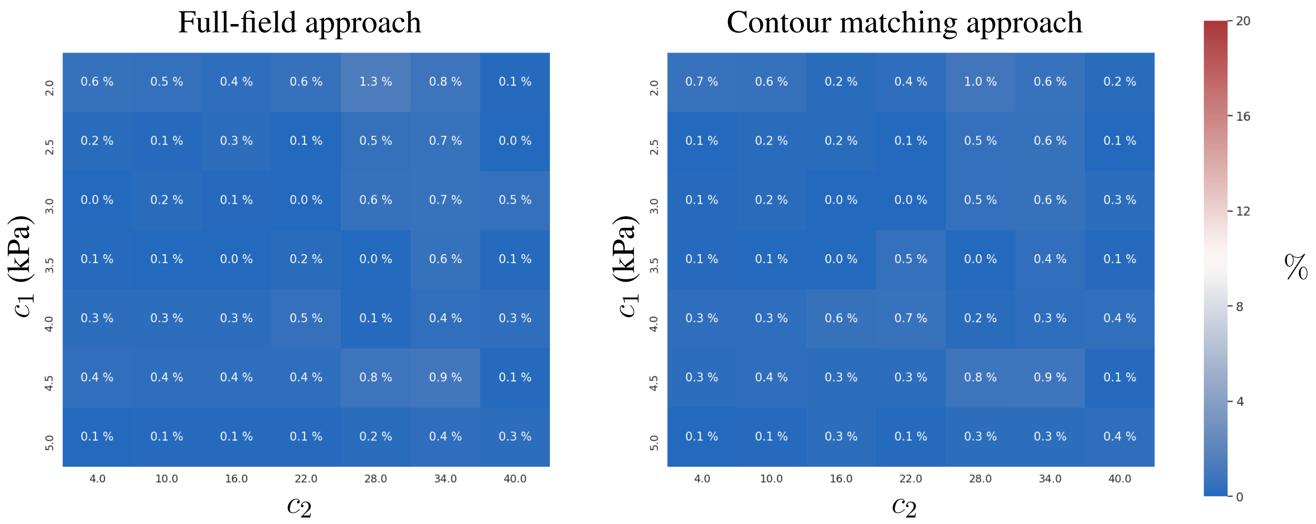

Figure S12. Relative error in estimating intra-ventricular pressure p using full-field tracking and contour matching approaches with a load of $p = 1.5$ kPa in the absence of noise in the observations.

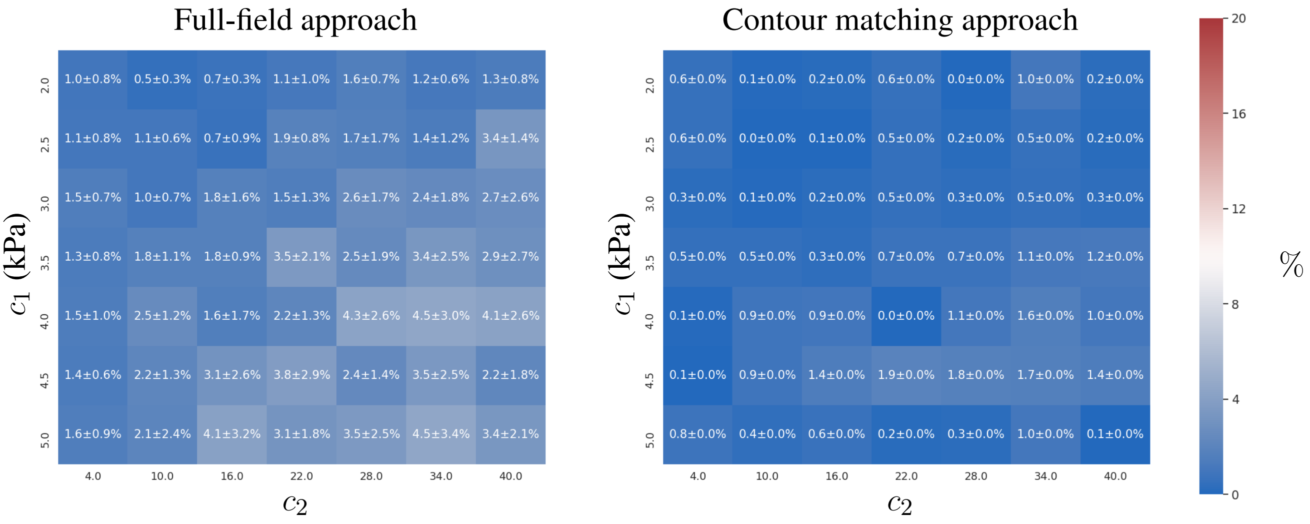

Figure S13. Mean and standard deviation of the relative errors for recovering ground truth intra-ventricular pressure using the full-field tracking and contour matching approaches and an intra-ventricular pressure of $p = 0.3$ kPa with Gaussian noise ($\sigma = 1\text{mm} \approx 2$ pixels) in the observations.

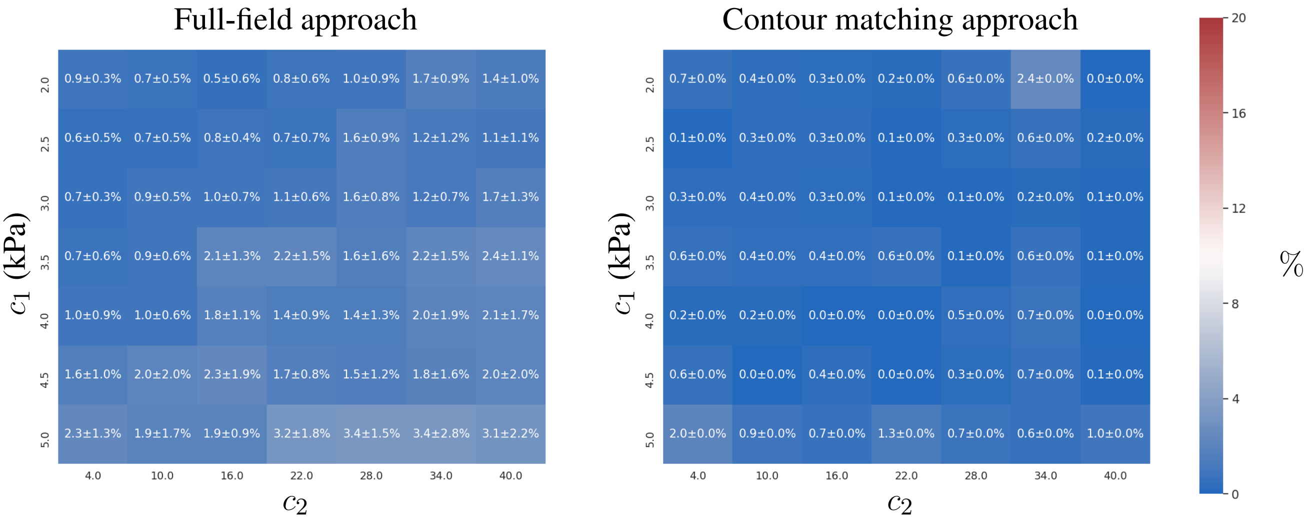

Figure S14. Mean and standard deviation of the relative errors for recovering ground truth intra-ventricular pressure using the full-field tracking and contour matching approaches and an intra-ventricular pressure of $p = 0.6$ kPa with Gaussian noise ($\sigma = 1\text{mm} \approx 2$ pixels) in the observations.

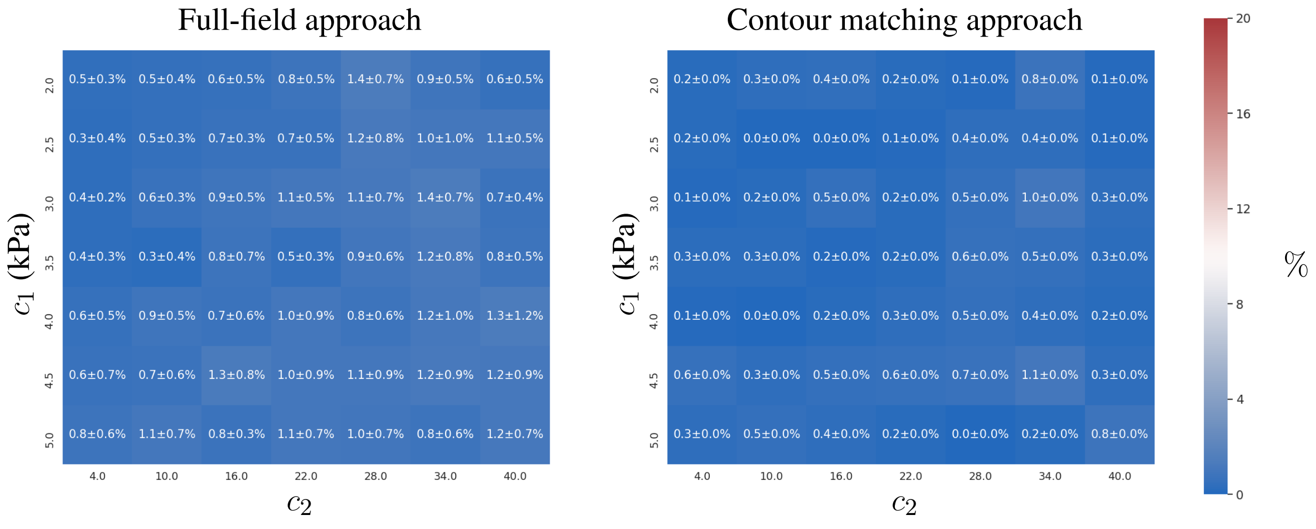

Figure S15. Mean and standard deviation of the relative errors for recovering ground truth intra-ventricular pressure using the full-field tracking and contour matching approaches and an intra-ventricular pressure of $p = 1.2$ kPa with Gaussian noise ($\sigma = 1$mm ≈ 2 pixels) in the observations.

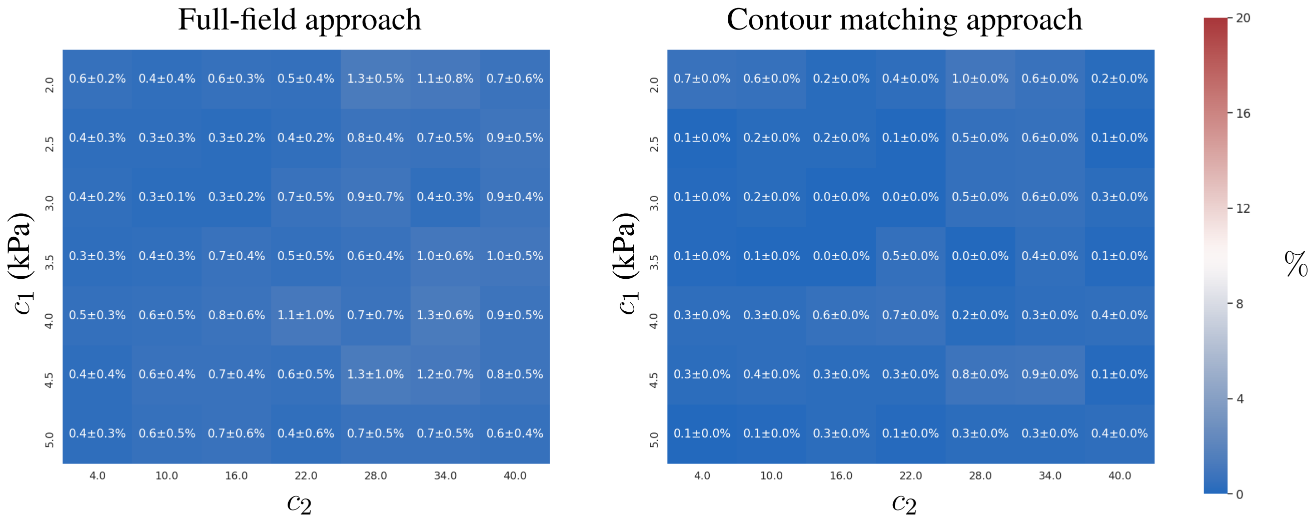

Figure S16. Mean and standard deviation of the relative errors for recovering ground truth intra-ventricular pressure using the full-field tracking and contour matching approaches and an intra-ventricular pressure of $p = 1.5$ kPa with Gaussian noise ($\sigma = 1$mm ≈ 2 pixels) in the observations.

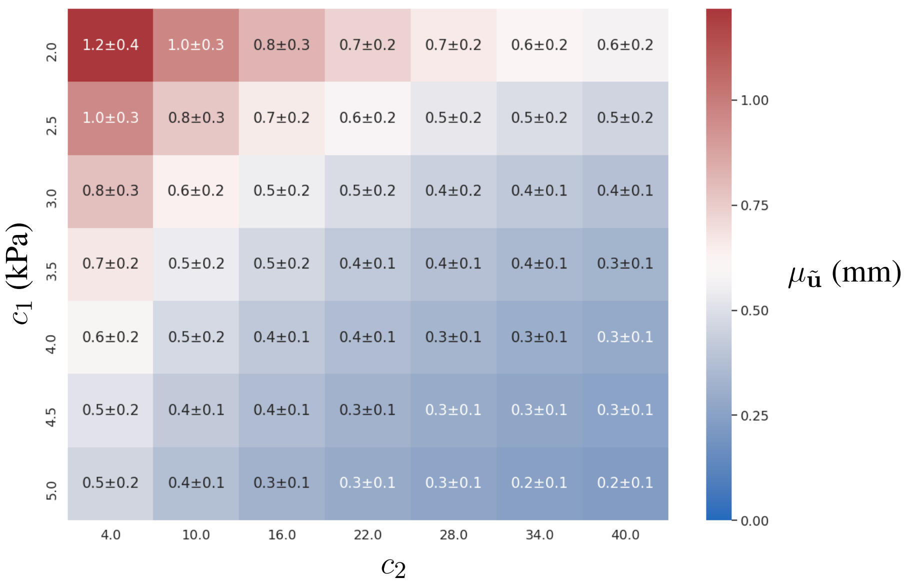

Figure S17. Mean displacement of the ventricular wall (denoted $\mu_{\hat{\mathbf{u}}}$) for different combinations of the constitutive parameters c_1 and c_2, and an intra-ventricular pressure load of $p = 0.3$ kPa.

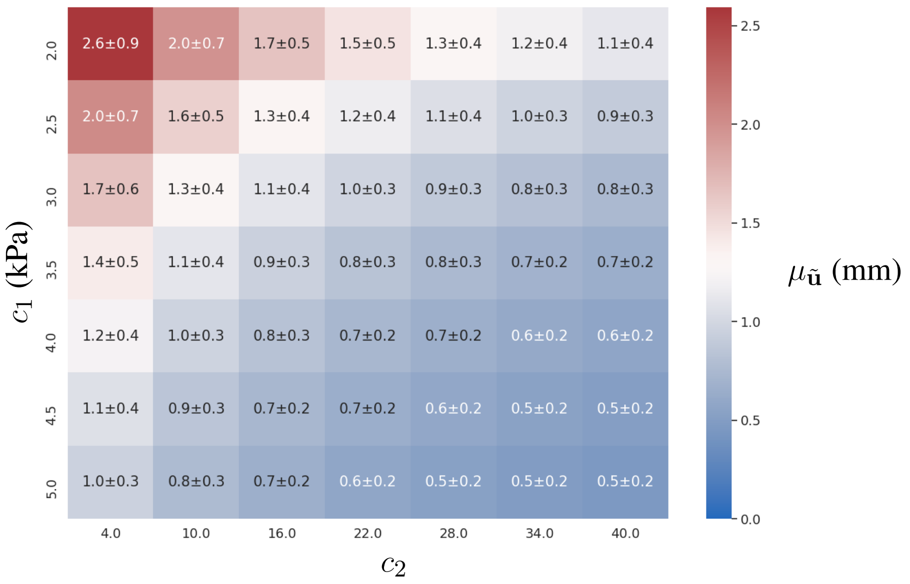

Figure S18. Mean displacement of the ventricular wall (denoted $\mu_{\hat{\mathbf{u}}}$) for different combinations of the constitutive parameters c_1 and c_2, and an intra-ventricular pressure load of $p = 0.6$ kPa.

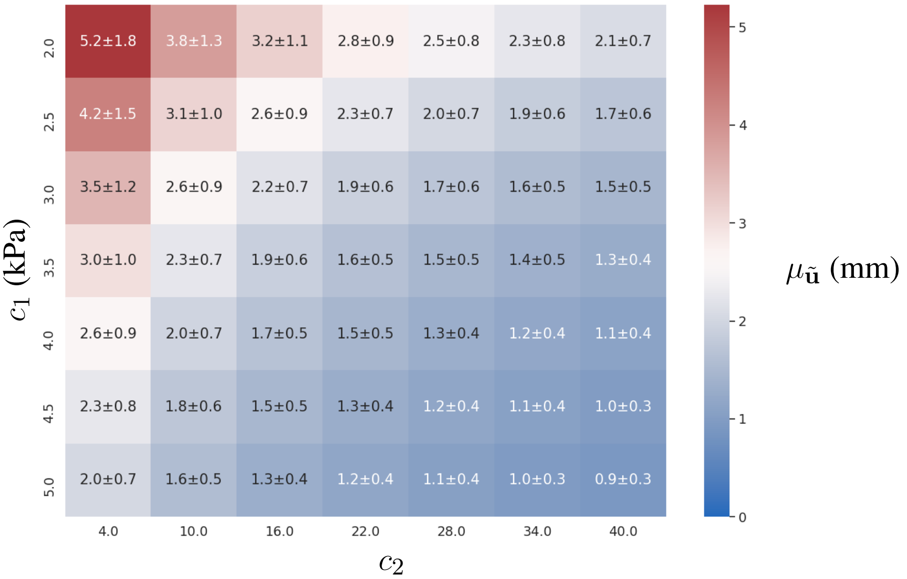

Figure S19. Mean displacement of the ventricular wall (denoted $\mu_{\hat{\mathbf{u}}}$) for different combinations of the constitutive parameters c_1 and c_2, and an intra-ventricular pressure load of $p = 1.2$ kPa.

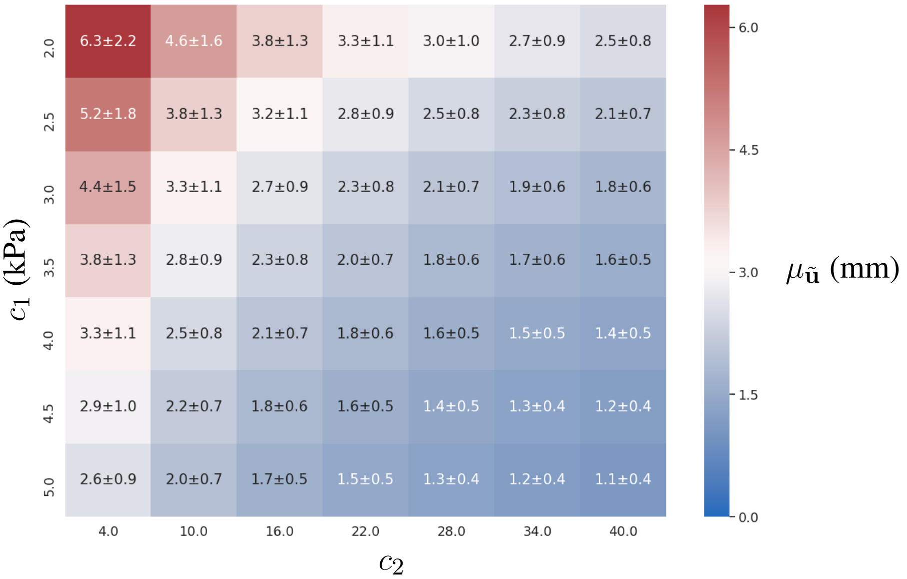

Figure S20. Mean displacement of the ventricular wall (denoted $\mu_{\hat{\mathbf{u}}}$) for different combinations of the constitutive parameters c_1 and c_2, and an intra-ventricular pressure load of $p = 1.5$ kPa.
